# Transcriptome analysis reveals genes associated with stem cell activation by physical exercise in the dentate gyrus of aged p16Ink4a knockout mice

**DOI:** 10.3389/fcell.2023.1270892

**Published:** 2023-10-19

**Authors:** Laura Micheli, Giorgio D'Andrea, Teresa Maria Creanza, Daniel Volpe, Nicola Ancona, Raffaella Scardigli, Felice Tirone

**Affiliations:** ^1^ Institute of Biochemistry and Cell Biology, National Research Council, Rome, Italy; ^2^ CNR-Institute of Intelligent Industrial Technologies and Systems for Advanced Manufacturing, Bari, Italy; ^3^ Institute of Translational Pharmacology, National Research Council, Rome, Italy; ^4^ European Brain Research Institute (EBRI), Rome, Italy

**Keywords:** adult neurogenesis, aging, dentate gyrus, neural stem cells, self-renewal, physical exercise, running, p16INK4a

## Abstract

Throughout adulthood neural stem cells divide in neurogenic niches–the dentate gyrus of the hippocampus and the subventricular zone–producing progenitor cells and new neurons. Stem cells self-renew, thus preserving their pool. Furthermore, the number of stem/progenitor cells in the neurogenic niches decreases with age. We have previously demonstrated that the cyclin-dependent kinase inhibitor p16Ink4a maintains, in aged mice, the pool of dentate gyrus stem cells by preventing their activation after a neurogenic stimulus such as exercise (running). We showed that, although p16Ink4a ablation by itself does not activate stem/progenitor cells, exercise strongly induced stem cell proliferation in p16Ink4a knockout dentate gyrus, but not in wild-type. As p16Ink4a regulates stem cell self-renewal during aging, we sought to profile the dentate gyrus transcriptome from p16Ink4a wild-type and knockout aged mice, either sedentary or running for 12 days. By pairwise comparisons of differentially expressed genes and by correlative analyses through the DESeq2 software, we identified genes regulated by p16Ink4a deletion, either without stimulus (running) added, or following running. The p16Ink4a knockout basic gene signature, i.e., in sedentary mice, involves upregulation of apoptotic, neuroinflammation- and synaptic activity-associated genes, suggesting a reactive cellular state. Conversely, another set of 106 genes we identified, whose differential expression specifically reflects the pattern of proliferative response of p16 knockout stem cells to running, are involved in processes that regulate stem cell activation, such as synaptic function, neurotransmitter metabolism, stem cell proliferation control, and reactive oxygen species level regulation. Moreover, we analyzed the regulation of these stem cell-specific genes after a second running stimulus. Surprisingly, the second running neither activated stem cell proliferation in the p16Ink4a knockout dentate gyrus nor changed the expression of these genes, confirming that they are correlated to the stem cell reactivity to stimulus, a process where they may play a role regulating stem cell activation.

## Introduction

In the adult brain, some areas retain the capacity of generating new neurons throughout an individual’s lifespan (Jurkowski et al., 2020). In particular, the two most studied neurogenic niches are the subgranular zone (SGZ) of the dentate gyrus of the hippocampus and the subventricular zone (SVZ) of the lateral ventricle (Doetsch et al., 1999; Seri et al., 2001; Obernier and Alvarez-Buylla, 2019). In the dentate gyrus, glia-like stem cells, called type-1, express Glial Fibrillary Acidic Protein (GFAP), Sex Determining Region Y-Box 2 (Sox2), and nestin, and mature into progenitor cells, classified as type-2a, positive for nestin and Sox2 (nestin^+^/Sox2^+^), type-2b positive for nestin and doublecortin (nestin^+^/DCX^+^) or type-3 (DCX^+^; Filippov et al., 2003; Fukuda et al., 2003; Kronenberg et al., 2003; Steiner et al., 2006). In turn, the progenitor cells develop into early post-mitotic cells (stage 5, co-expressing DCX and NeuN) and then into fully differentiated neurons (stage 6, expressing calbindin and NeuN) (Brandt et al., 2003; Steiner et al., 2004).

During aging both neurogenic niches exhibit a decline in the ability of stem cells to self-renew and produce new neurons (Kuhn et al., 1996; Bizon and Gallagher, 2003; Bondolfi et al., 2004; Enwere et al., 2004; Maslov et al., 2004; Couillard-Despres et al., 2009). In the SGZ, this is accompanied by a reduced performance of memory tasks dependent on the hippocampus (van Praag et al., 2005).

Moreover, it is known that following the removal of cell cycle inhibitors, such as p21 or Btg1, dentate gyrus stem and progenitor cells gradually lose their capacity to proliferate and self-renew (Kippin et al., 2005; Farioli-Vecchioli et al., 2012). This underscores the pivotal importance of cell cycle regulation in sustaining self-renewal.

In this regard, a critical role in aging and senescence is played by the cell cycle inhibitor *p16 Ink4a* (Rayess et al., 2012) (hereafter called p16), which binds to CDK4 and CDK6, inhibiting the phosphorylation of the retinoblastoma suppressor (Rb) and the subsequent start of the G1/S cell cycle checkpoint (Serrano et al., 1993; Hannon and Beach, 1994). The expression of p16 increases with age in mouse tissues and becomes noticeable within the neurogenic niches at 1 year of age (Molofsky et al., 2006; Micheli et al., 2019).

Furhermore, p16 negatively controls SVZ neurogenesis during aging. In fact, the knockout of p16 reverts the age-dependent loss of self-renewal in the aging SVZ; yet, notably, no effect on neural cell self-renewal or proliferation is seen in the adult and aging p16 knockout dentate gyrus (Molofsky et al., 2006).

Nevertheless, we have recently shown that in the dentate gyrus of aging (1-year-old) p16 knockout mice the number of proliferating stem cells (type-1) and early progenitor cells (type-2a) is greatly enhanced by voluntary physical exercise (running), without any effect on the stem cells of wild-type mice (Micheli et al., 2019). The majority of the newly formed stem and progenitor cells developed into neurons, demonstrating that in p16 knockout mice the new stem cells were capable of growth and were not restricted solely to self-renewal, although part of them underwent apoptosis. Furthermore, after the neurogenic stimulus of running was arrested, the new stem and progenitor cells continued to actively proliferate in p16 knockout mice for a longer period of time than in wild-type mice, demonstrating that stem cell reactivation was sustained (Micheli et al., 2019). Therefore, p16 prevents the activation of dentate gyrus stem cells by a stimulus during aging, thereby preserving the pool (Micheli et al., 2019). On the other hand, it is known that the increased expression of p16 drives the stem cells into senescence, with a terminal exit from the cell cycle (Rayess et al., 2012).

The activation of neurogenesis/self-renewal in the p16 knockout dentate gyrus by running is in line with our previous observations that a neurogenic stimulus (running or other types of stimuli) can reactivate neural stem cells whose proliferative potential has been reduced as consequence of aging or deletion of a cell cycle inhibitor, such as Btg1 (Farioli-Vecchioli et al., 2014; Micheli et al., 2018a; D’Andrea et al., 2020). All this suggests that neural stem cells of adult neurogenic niches have a reserve of proliferative capability exploitable during aging or throughout life upon stimulation **(** Bonaguidi et al., 2011; Farioli-Vecchioli et al., 2014; Mastrorilli et al., 2017; Micheli et al., 2018a; Ceccarelli et al., 2020), rather than being subjected to progressive, complete depletion (Martín-Suárez and Encinas, 2021).

Although running is capable to counteract the decrease in neurogenesis during aging (van Praag et al., 2005; Marlatt et al., 2012), as it induces the proliferation of hippocampal adult progenitor cells (Ryan and Kelly, 2016) and SVZ neuroblasts (Bednarczyk et al., 2009), is nevertheless unable to activate wild-type stem cells in the dentate gyrus (Kronenberg et al., 2003; Brandt et al., 2010; Micheli et al., 2017). We concluded that the activation of stem cells in p16 knockout dentate gyrus by running indicates that p16 prevents neural stem and progenitor cells from responding to a neurogenic stimulus, thereby preserving the stem cell pool and its capacity for self-renewal during aging (Micheli et al., 2019).

To better define the role of p16 in the maintenance of the stem cell pool during aging, in this report we analyzed by RNA sequencing the whole transcriptome of the neurogenic niche of dentate gyrus, in p16 knockout and wild-type aging mice, subjected to voluntary running or sedentary conditions.

Through pairwise comparisons of differentially expressed genes and by software-assisted correlative analyses, we identified genes that represent either the basic signature of p16 knockout (i.e., in sedentary mice) or whose expression matches the previously observed pattern of activation of stem cells by running. The two gene ensembles do not overlap, except marginally, suggesting that the p16 knockout genetic signature and stem cell activation by running in p16 knockout dentate gyrus involve different neural processes.

Moreover, to investigate the plasticity of the process of neural cell self-renewal in the SGZ and the role of p16 in this process, we have experimentally tested the resilience of the activation response in stem cells to sequential running stimuli, in correlation with the stem cell-specific gene regulation; for this purpose, we subjected p16 wild-type and knockout mice to two consecutive voluntary running sessions, spaced 3 weeks apart. It turned out that the second stimulus, unlike the first, failed to induce a proliferative response in p16 knockout stem cells. This suggests that the process of stem cell activation is strictly regulated and/or undergoes exhaustion. The expression of the genes that we found to be regulated concomitantly with stem cell activation in p16 knockout mice is not changed or is counter-regulated after the second stimulus, further indicating that they play a role in stem cell activation.

## Materials and methods

### Mouse line, genotyping and husbandry

The *p16Ink4a* knockout (p16 KO) and *p16Ink4a* wild-type (p16 WT) mouse lines in the C57BL/6 background were obtained as previously described (Micheli et al., 2019). The C57BL/6 mouse strain was chosen as is frequently used for research on neurogenesis (Molofsky et al., 2006; Kim et al., 2017). Briefly, the *p16Ink4a* knockout was previously generated as a homozygous knockout mouse in FVB background (Sharpless et al., 2001) and was obtained from the Frederick National Laboratory for Cancer Research (strain number 01XE4; Frederick, MD, USA). This strain has functional *p19Arf* despite having a null allele of the *p16Ink4a* gene. Then, by breeding *p16Ink4a+/-* mice with the C57BL/6 strain for at least six generations, until an isogenic progeny was established, *p16Ink4a* knockout and p16Ink4a wild-type strains with a C57BL/6 background were generated (also referred to as p16 KO and p16 WT throughout the paper).

Regarding the predisposition to tumorigenesis in p16 KO mice (Sharpless et al., 2001), in 1-year old p16KO mice we did not observe evident difference in survival relative to wild-type, which is in agreement with Sato et al. (2015). Any p16 KO mice exhibiting noticeable physical or behavioral abnormalities were excluded from the study.

Genotyping was routinely performed by PCR analysis, using genomic DNA from tail tips as previously described (Farioli-Vecchioli et al., 2012). The primers used for genotyping were: C015: 5′-ggc​aaa​tag​cgc​cac​cta​t-3'; C016: 5′-gac​tcc​atg​ctg​ctc​cag​at-3'; C017: 5′-gcc​gct​gga​cct​aat​aac​ttc-3' (Micheli et al., 2019).

Mice were maintained under standard specific-pathogen-free conditions and were housed in standard cages until they reached 1 year of age. Then, mice were randomly assigned to either a standard cage or one containing a running wheel. Wheel rotations were recorded daily with an automatic counter. The body weights of the mice were also recorded and no significant differences related to the genotype or treatment were observed (data not shown).

All animal procedures were performed on male mice and completed in accordance with the current European (Directive 2010/63/EU) Ethical Committee guidelines and the protocol of the Italian Ministry of Health (authorizations 1209/2015-PR and 550/2022-PR).

### Running protocol for p16 KO and p16 WT mice used for RNA-seq analysis and real-time PCR validation

**FIGURE 1:**
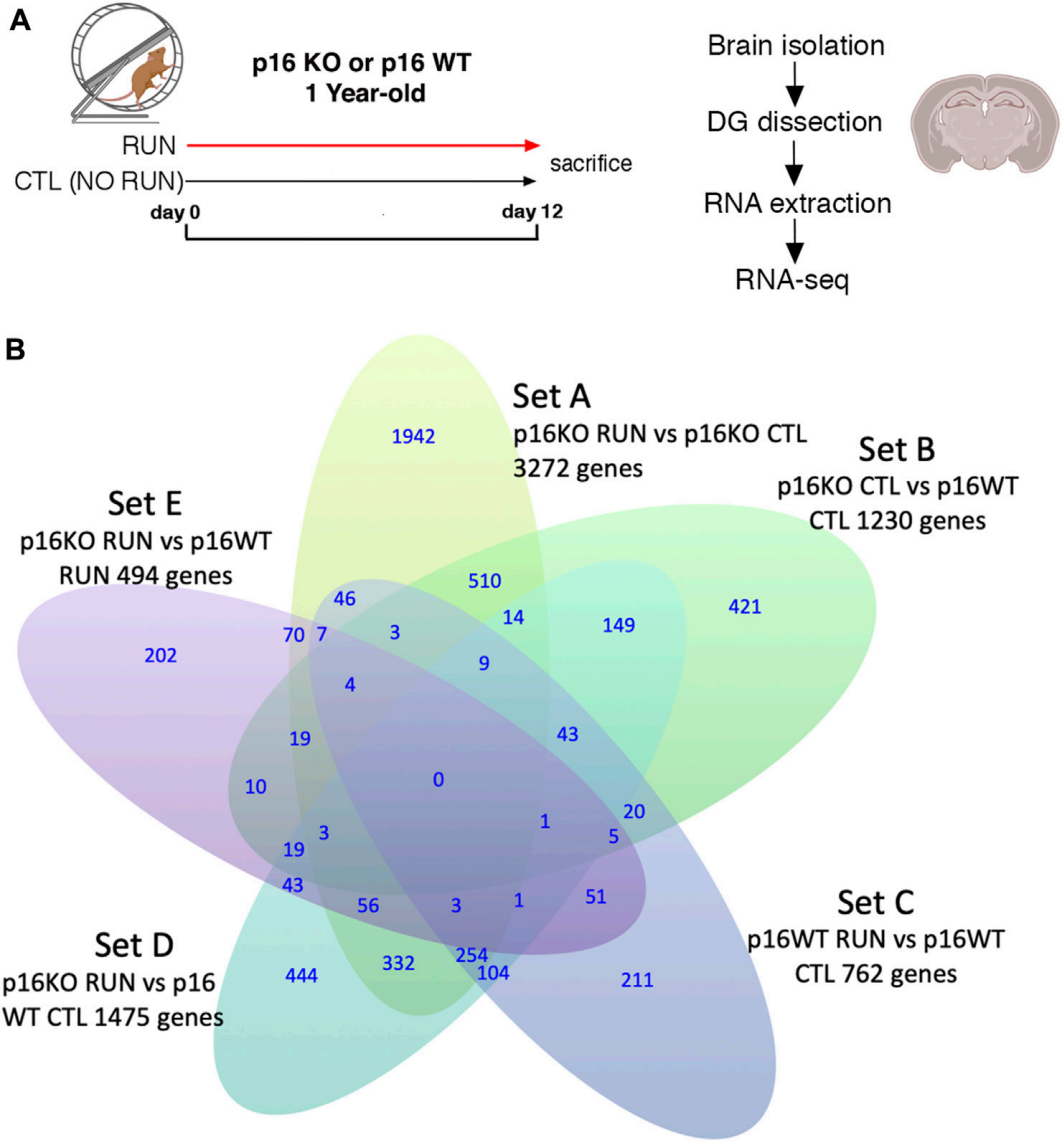
**(A)** Timeline of voluntary running exercise with experimental scheme of RNA seq. **(B)** Venn Diagram indicating five pairwise comparisons (i.e., sets) of genotypes (p16 KO and p16 WT) vs treatment (RUN and CTL) relative to the expression of genes in the dentate gyrus, as well as the intersection of the differentially expressed gene sequences in sets A-E. Set A corresponds to the pairwise comparison p16 KO RUN vs p16 KO CTL; set B indicates p16 KO CTL vs p16 WT CTL; set C represents p16 WT RUN vs p16 WT CTL, set D corresponds to the comparison p16 KO RUN vs p16 WT CTL, and set E to the comparison p16 KO RUN vs p16 WT RUN. The genes showing significant pairwise differential expression were identified by the DESeq2 software using the Wald test (Love et al., 2014), with significance threshold of the log_2_ fold change *p*-value <0.05. RNA seq results were obtained from four independent samples for each of the four experimental groups (two mice per sample).

For RNA analyses, one-year-old mice were allowed to run for 12 days and then were euthanized to dissect the dentate gyrus (see the experimental timeline in Figure 1A).

The average running wheel distance over the 12-day-experiment was 2.71 km/day ±0.67 Standard Error Mean (SEM) for p16 WT mice and 2.98 km/day ±0.92 (SEM) for KO mice, without significant differences (*p* = 0.86, Student’s t-test); the total distances run were on average 32.54 km ± 8.00 (SEM) for p16 WT and 35.74 km ± 11.03 (SEM) for p16 KO mice, *p* = 0.86; n p16 WT mice = 8, n p16 KO mice = 8, Student’s t-test.

### Running protocol for p16 KO and p16 WT mice used for immunohistochemistry analyses and real-time PCR

For immunohistochemistry analyses and Real-Time PCR (RT-PCR) studies, one-year-old mice had access to the running wheel for 12 days and/or 7 days, or were housed in standard cages (sedentary, NO RUN) and were sacrificed after the end of the experimental protocol, as indicated (see experimental timelines in Figure 5A).

When compared, p16 WT and p16 KO mice did not show significant differences in 12 days running performances, as measured in the different protocols. The average running wheel distance was 2.17 km/day ±0.26 (SEM) for p16 WT mice and 2.56 km/day ±0.39 (SEM) for p16 KO mice (*p* = 0.42, Student’s t-test); the total distances run were on average 26.07 km ± 3.21 (SEM) for p16 WT and 30.81 km ± 4.78 (SEM) for p16 KO mice (*p* = 0.42, p16 WT mice n = 11, p16 KO mice n = 11, Student’s t-test).

Analogously, no significant differences were observed between p16 WT and p16 KO mice that were exposed to the running wheel for 7 days (in different protocols). The average running wheel distance was 1.81 km/day ±0.34 (SEM) for p16 WT mice and 2.34 km/day ±0.39 (SEM) for p16 KO mice (*p* = 0.31, Student’s t-test); the total distances run were on average 12.68 km ± 2.36 (SEM) for p16 WT and 16.38 km ± 2.74 (SEM) for p16 KO mice (*p* = 0.31, p16 WT mice n = 11, p16 KO mice n = 11, Student’s t-test).

### Dentate gyrus dissection and RNA isolation

One-year-old p16 WT and p16 KO mice, sedentary, 12 days runner (for RNA-seq) or subjected to a further 7 days of running (protocol 12d RUN +21d + 7d RUN in Figure 5A), were sacrificed by rapid decapitation. The bilateral dentate gyri were dissected under a stereomicroscope according to the procedure described by Hagihara et al. (2009), immediately homogenized in TRIzol Reagent (Invitrogen, San Diego, CA, USA). Subsequently, total RNA extraction was performed as described previously (Farioli-Vecchioli et al., 2012). Extracted RNA was quantified and assessed for purity using a NanoDrop ND-1000 Spectrophotometer (Thermo Fisher Scientific, Wilmington, DE, USA) and an Agilent 2,100 bioanalyzer (Agilent Technologies, Santa Clara CA). The RNAs were subsequently employed for Transcriptome sequencing and/or for real-time PCR experiments.

### Transcriptome sequencing

RNA-sequencing was performed using total RNA isolated from the dentate gyrus of one-year-old p16 wild-type or knockout mice, either submitted to running for 12 days or sedentary (8 animals per group). Four independent samples were used in total for each of the four experimental groups, and each sample was obtained by pooling together the dentate gyri from two mice.

The purified RNA was sent to IGA Technology Services (https://www.igatechnoloy.com) for RNA library preparation using a universal Plus mRNA-Seq kit (Tecan Genomics, Redwood City CA, USA, following the manufacturer’s instructions). Final libraries were checked with both Qubit 2.0 Fluorometer (Invitrogen, Carlsbad, CA, USA) and by Caliper LabChip GX (PerkinElmer, Waltham, MA). RNA sequencing was performed by NovaSeq 6,000 (Illumina, San Diego, CA) through paired-end 150 bp reads, and 80M reads on average per sample, followed by standard bioinformatic analysis.

This consisted of processing raw data for base calling and demultiplexing by Bcl2Fastq 2.20 version of the Illumina pipeline; adapters masking with Cutadapt v1.11; trimming of lower quality bases and adapters by ERNE software; aligning reads on reference mm10-ucsc genome/transcriptome with STAR; executing transcripts count by Stringtie; quality control by the RSeqQC package.

This was followed by pair-wise differential expression analysis: initially htseq-count (Anders et al., 2015) was used to preprocess RNA-Seq data for differential expression analysis by counting the overlap of reads with genes. DESeq2 (Anders and Huber, 2010; Love et al., 2014) was then used to perform comparisons between expression levels of genes and transcripts in two different conditions. Briefly, DESeq2 models the raw counts, using normalization factors (size factors) to account for differences in library depth. Then, it estimates the gene-wise dispersions and shrinks these estimates to generate more accurate estimates of dispersion to model the counts. Finally, DESeq2 fits the negative binomial model and perform hypothesis testing using the Wald test, which gives the probability that the observed differences of gene expression are significantly different than by chance (Love et al., 2014).

We used also a method included in the DESeq2 package, preliminary or alternative to pair-wise comparisons, by fitting a Generalized Linear Model (GLM) for each gene that accounts for all levels of a factor and its interactions at once and uses the Likelihood ratio test (LRT). LRT method makes a ratio of likelihood estimates between a “full” model, which comprises the full variance inherent to all factors (in our case genotype and treatment) including their interaction (genotype vs treatment), and a “reduced” model that comprises the full variance of factors except their interaction. The *p*-values are determined by the difference in deviance between the ‘full’ and ‘reduced’ model formula, allowing to calculate by difference the interaction between factors, even in a multi-level experiment, and to identify all differentially expressed (DE) genes with significant differences (i.e., interaction between the two levels) in the effect of treatment over genotype.

Moreover, the RNA sequencing datasets are deposited at the Gene Expression Omnibus (GEO) repository with Accession Number GSE237736 (https://www.ncbi.nlm.nih.gov/geo/).

### Gene Ontology enrichment

We carried out a Gene Ontology (GO) enrichment analysis with the aim to discover GO terms that were significantly over-represented in genes that were differentially regulated in specific comparisons, and then, to explore possible functional properties of these genes. It is possible to infer regulatory mechanisms or functional pathways that are activated or repressed in the analyzed conditions by looking at the GO terms enriched in the set of genes that are significantly differentially expressed.

We used the GO annotations retrieved from the Molecular Signatures Database (MSigDB) web site (https://www.gsea-msigdb.org/gsea/msigdb/) for the *Homo sapiens* species. This web site was also used to compute overlaps between our set of differentially regulated genes and gene sets in MSigDB. In detail, the *p*-values for enrichment are determined by the cumulative distribution function of the hypergeometric distribution (Fisher’s test), and the provided false discovery rate (FDR) q-values are an analog of the hypergeometric *p*-value after correction for multiple hypothesis testing according to Benjamini and Hochberg. Regarding the genes counter-regulated in SetB with respect to the CellAge senescence gene database (https://genomics.senescence.info/cells/signatures.php), their GO enrichment analysis was performed using the GSEA software applied to the existing Gene Ontology annotations for *Mus musculus*. The *p*-values for enrichment were calculated by Fisher’s exact test using the GSEA software.

### Real-time PCR

To validate the RNA sequencing results, total RNA extracted from the isolated dentate gyri was reverse-transcribed as previously described (Farioli-Vecchioli et al., 2012). The same samples used for RNA sequencing were employed for analysis in each of the four groups (four samples per group). Each sample consisted of dentate gyri from two mice.

Analogously, total RNA was extracted and reverse-transcribed for analysis of expression in dentate gyrus of p16 WT and p16 KO mice subjected to the protocol of double running (12d RUN +21d +7d RUN). Five samples per group were used.

The Applied Biosystems’ 7900HT System was used to perform real-time PCR on triplicate samples using SYBR Green I dye chemistry. Relative quantification was performed by the comparative cycle threshold method (Livak and Schmittgen, 2001). The mRNA expression levels were normalized to those of the TATA-binding protein (TBP) gene set as endogenous control. Statistical analysis of mRNA expression values was performed by Mann-Whitney U test on the RQ values of each comparison, after the Kruskal-Wallis test to analyze the main effects in the different groups together (i.e., p16 WT and KO, either CTL and RUN, or RUN and double RUN). TBP sequences were: TBP-F: 5′ CCA​ATG​ACT​CCT​ATG​ACC​CCT​A-3′ and TBP-R: 5′-CAG​CCA​AGA​TTC​ACG​GTA​GAT-3’. Average ±SEM values of fold-changes relative to the control sample are shown. Specific RT-PCR primers used were deduced from published murine cDNA sequences; their sequence is available upon request.

### Immunohistochemistry

The mouse brains were collected after transcardiac perfusion with 4% paraformaldehyde (PFA) in PBS 1x and kept overnight in 4% PFA. Brains were then equilibrated in 30% sucrose and cryopreserved at −80°C. Immunohistochemistry was performed on serial free-floating coronal sections cut at 40 μm thickness in a cryostat at −25°C from brains embedded in Tissue-Tek OCT (Sakura Finetek, Torrance, CA, USA).

Proliferating stem and progenitor cells were visualized by means of a rabbit monoclonal antibody against Ki67 (Invitrogen, San Diego, CA, USA; MA514520; 1:200), a goat polyclonal antibody against Sox2 (Abcam, Cambridge, UK; Ab239218; 1:300), and a mouse monoclonal antibody against GFAP (Sigma-Aldrich, St Louis, MO; G6171; 1:200). Sections were previously permeabilized with 0.3% TritonX-100 in PBS, and then incubated with primary antibodies against Ki67 and Sox2 with 3% normal donkey serum in 0.3% TritonX-100 in PBS for 16–18 h at 4°C, followed by incubation with secondary antibodies. Sections were post-fixed with 4% paraformaldehyde (PFA) for 10 min and then incubated with the anti-GFAP antibody with 3% normal donkey serum in 0.3% TritonX-100 in PBS for 16–18 h at 4°C, followed by incubation with the secondary antibody.

Secondary antibodies used to visualize the antigen were from Jackson ImmunoResearch (West Grove, PA, USA) as follows: a donkey anti-rabbit antiserum conjugated to tetramethylrhodamine isothiocyanate (TRITC) (Ki67) and a donkey anti-goat antiserum conjugated to Alexa-647 (Sox2); a donkey anti-mouse antiserum conjugated to Alexa-488 (GFAP), incubated for 1 h. At the end of the procedure, to reduce auto-fluorescence of the tissue, slices were treated with 0.3% Sudan Black (Sigma-Aldrich, St Louis, MO) in 70% ethanol for 30 s and rinsed thoroughly with PBS 1x. Nuclei were counterstained by Hoechst 33,258 (Sigma–Aldrich, St Louis, MO; 1 μg/ml in PBS).

Confocal Z-stacks and single plane-images of the immunostained sections were obtained using a TCS SP5 confocal laser scanning microscope (Leica Microsystem, Wetzlar, Germany).

### Quantification of cell numbers

The cells positive for the indicated markers were counted throughout the whole rostrocaudal extent of the dentate gyrus, in one-in-six series of 40-μm free-floating coronal sections (240 μm apart), analyzed with confocal microscopy. To obtain the total estimated number of positive cells within the whole dentate gyrus, the average number of positive cells per section was multiplied by the total number of sections including the entire dentate gyrus (approximately 50–60 sections), as described (Farioli-Vecchioli et al., 2008; Jessberger et al., 2005; see also Noori and Fornal, 2011 regarding the cell counting theory). Approximately 8–10 sections (16–20 dentate gyri areas) per mouse and at least three animals per group were analyzed. Cell number analyses were performed manually by trained experimenters, in blinded fashion, using the IAS software to register positive cells (Delta Sistemi, Rome, Italy).

### Statistical analyses

**FIGURE 2:**
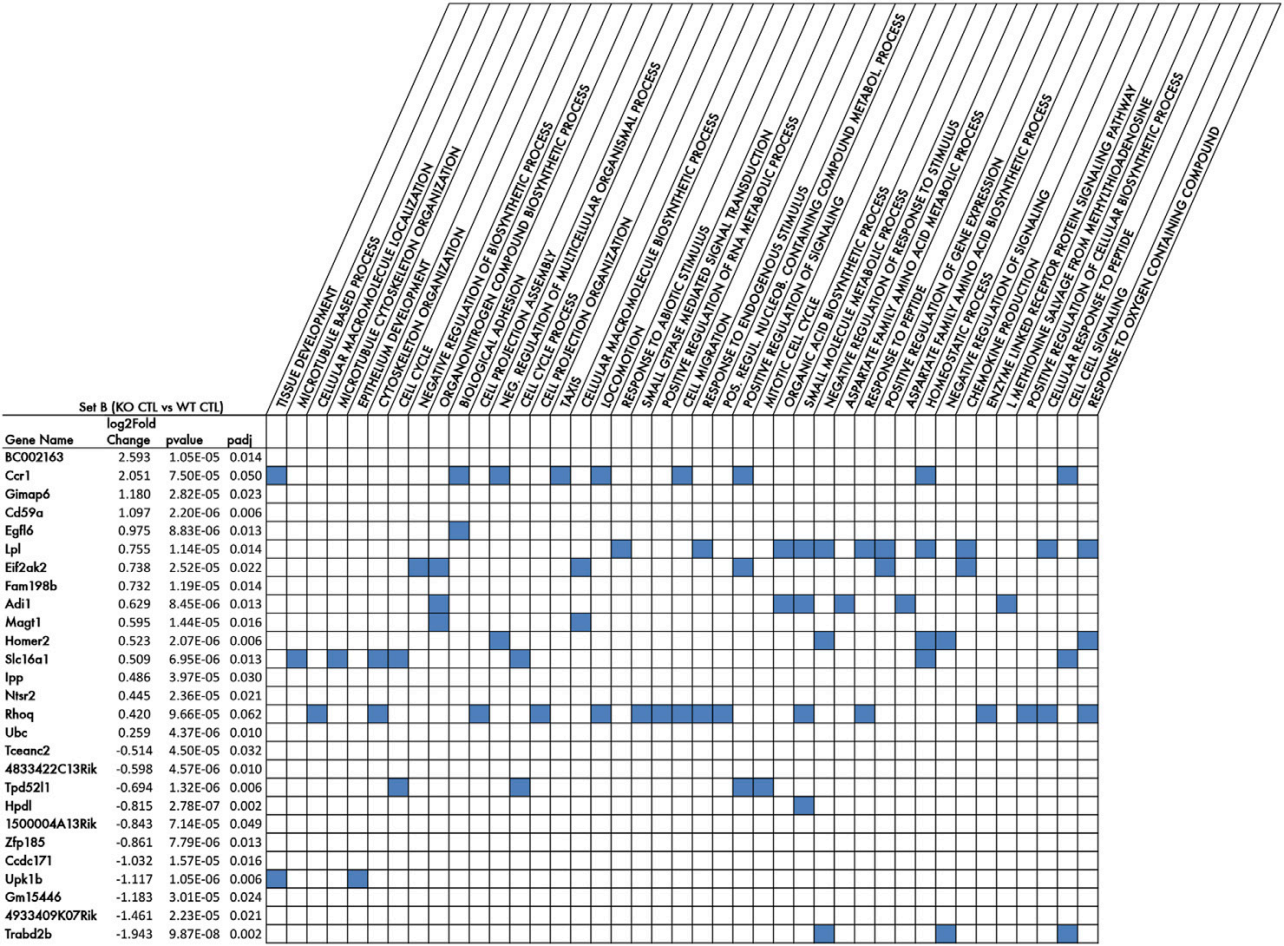
Top DE genes of Set B comparison, i.e., the genetic signature of p16 knockout in aged dentate gyrus, and the biological processes enriched in these genes (GOBP terms). Listed in rows are the 27 genes showing the top significant differences in expression between the two groups of the Set B comparison, i.e., p16KO CTL vs p16WT CTL, (differentially expressed genes with log2fold change *p*-value **≤** 0.0001) sorted by decreasing log2 Fold change. The pairwise calculations of differential gene expression and their statistical significance were performed by DESeq2 (Love et al., 2014). The full list of DE genes of Set B comparison is shown in Supplementary Table S1. Log2 Fold change is the expression fold change occurring for each gene within the Set B comparison; *p*-value is the probability generated by the Wald test (Love et al., 2014), under the null hypothesis, of obtaining the Set B log2 Fold change observed; padj is the *p*-value adjusted for multiple testing with the Benjamini-Hochberg procedure to control the false discovery rate (FDR). Columns show the GO Biological Processes significantly enriched (FDR q-value <0.05) of Set B genes DE with Wald test *p*-value <0.01 and including the top DE genes indicated in the rows. The full list of the significantly enriched (FDR q-value <0.05) GO Biological Processes is shown in Supplementary Table S2 **.**

The Wald test *p*-value (p) (part of the DESeq2 package; Love et al., 2014; Anders and Huber, 2010) was used to perform the pairwise comparison of differential gene expression, i.e., the comparison of the mean fold-expression changes between samples of two groups from the different data sets (*n* = 4 samples per group); *p*-value was corrected for False Discovery Rate (FDR) to obtain the *padj*-value, i.e., *p*-value adjusted for multiple hypothesis testing using the procedure of Benjamini and Hochberg (Love et al., 2014; Figure 1, Supplementary Table S1; Figure 2, Supplementary Table S4; Table 1, Supplementary Table S5; Table 2, Supplementary Table S6).

**TABLE 1:** Focused search for genes correlated to the proliferative activation of stem cells: selection of the differentially expressed GLM genes whose expression was significantly changed in the dentate gyrus by running in both Set D and Set E (termed Set D_Set E_GLM). The genes identified by the GLM procedure, i.e., whose expression was significantly changed by interaction between running and genotype, were further selected for their expression being induced or reduced by running with *p* < 0.05 in the pairwise comparisons of Set D (p16 KO RUN vs p16 WT CTL) and of Set E (p16 KO RUN vs p16 WT RUN). In this way 29 genes in total were selected (Set D_Set E_GLM selection). The regulation of these genes matches the pattern of activation of p16 KO stem cells by running (see Micheli et al., 2021), with maximal differential change of expression in the pairwise comparisons analyzed (i.e., with p16 WT CTL or with p16 WT RUN). Among Set D_Set E_GLM genes we show here those having a function that, according to the current literature, can be connected with the activation of neurogenesis, i.e., of stem and progenitor cells of the dentate gyrus, ranked by decreasing log2 fold change in Set D. The full list of 29 genes of Set D_Set E_GLM is shown in Supplementary Table S5. The genes written in red are those with significant differential expression also in Set B (p16 KO CTL vs p16 WT CTL groups). p-values for Set D and Set E genes were obtained by DESeq2 analysis through the Wald test, while p-values for genes selected by the GLM model were obtained by testing the difference between the ‘full’ and ‘reduced’ model (Love et al., 2014).

	Set D		Set E		GLM		
	KO RUN vs WT CTL		KO RUN vs WT RUN				
Gene name	log2Fold change	P-value	log2Fold change	P-value	P-value	Gene function correlated to stem cell activation	References
Mki67	1.3571	0.00000003	1.48700	0.0000212	0.00055784	Cell cycle regulator	Scholzen and Gerdes (2000)
Top2a	1.2416	0.0006039	1.44020	0.0000284	0.0081051	Cell cycle regulator	Gómez-Herreros et al. (2014)
Lepr	1.1527	0.000036	0.80141	0.001078	0.0041875	Synaptic plasticity regulator	Dhar et al. (2014)
Zic4	0.9007	0.04344	0.91747	0.042865	0.04725	Activator of stem cell proliferation	Blank et al. (2011)
Nlrc5	0.8740	0.014378	0.91737	0.045703	0.0014689	Negative ROS regulator	Li et al. (2021a)
Slc18a2	0.8177	0.0022055	0.86778	0.0011748	0.016933	Synaptic plasticity regulator	Stefanovic et al. (2016)
Prc1	0.7320	0.049167	1.15650	0.0027045	0.0083644	Cell cycle regulator	Mollinari et al. (2002)
Gstm2	0.6531	0.040371	0.81297	0.0047004	0.021936	Negative ROS regulator	Segura-Aguilar et al. (2014)
Pole	0.6457	0.00032249	0.34811	0.039086	0.011691	Cell cycle regulator	Hogg and Johansson (2012)
Cenpf	0.6309	0.0052725	0.83230	0.0001357	0.0014211	Synaptic plasticity regulator	Tanaka et al. (2012)
Unc13d	0.5407	0.04825	0.46491	0.024812	0.025937	Synaptic plasticity regulator	Dittman (2019)
Mocos	0.4824	0.046743	0.37830	0.020257	0.015645	Negative ROS regulator	Féron et al. (2016)
Lpin2	0.2358	0.0000206	0.17873	0.015751	0.00075486	Fatty acid metabolism regulator	Chen et al. (2015)
Gabbr2	0.1696	0.0010229	0.13118	0.024323	0.0091136	Synaptic plasticity regulator	Heaney and Kinney (2016)
Cnbp	−0.1752	0.001746	−0.14838	0.0061765	0.031318	Cell cycle regulator	Calcaterra et al. (2010)
Rgs14	−0.2057	0.018748	−0.22733	0.0043943	8.33E-05	Synaptic plasticity negative regulator	Evans et al. (2018)
Ttc9	−0.2738	0.031716	−0.33005	0.01287	0.002817	Cell division?	Cao et al. (2008)
Ramp3	−0.5486	0.0086496	−0.60432	0.018916	0.015335	Pro-differentiative	Li et al. (2021b)

**TABLE 2:** Wide search for genes correlated to the proliferative activation of stem cells: selection of the 866 differentially expressed GLM genes whose expression was significantly changed in the dentate gyrus by running in both Set A and Set E (termed Set A_Set E_GLM). This analysis is parallel to, but wider than, the one shown for Set D_Set E_GLM in Table 1. In fact the 866 genes identified by the GLM procedure, i.e., whose expression was significantly changed by the interaction between running and genotype, were further selected for genes changed by running with *p* < 0.05 in both pairwise comparisons of Set A (p16 KO RUN vs p16 KO CTL) and of Set E (p16 KO RUN vs p16 WT RUN). The total number of genes identified in this way was 106: this group was named Set A_Set E_GLM selection and its differential gene regulation reproduces the pattern of proliferative activation of p16 KO stem cells by running, with respect to the other groups. Moreover, Set A_Set E_GLM selection includes all genes of Set D_Set E_GLM shown in Table 1, indicating that the Set A_Set E_GLM is more comprehensive. The list shown here is a selection among the Set A_Set E_GLM genes of those that could be functionally correlated, according to the existing literature, to the activation of stem and progenitor cells of the dentate gyrus, sorted by decreasing log2 fold change in Set A. The full list of 106 genes of Set D_Set E_GLM is shown in Supplementary Table S6. The genes highlighted in red are those with significant differential expression also in Set B (p16 KO CTL vs p16 WT CTL groups), i.e., the set representing the genetic signature of p16 knockout. p-values for Set A and Set E genes were obtained by DESeq2 analysis through the Wald test, while p-values for genes selected by the GLM model were obtained by testing the difference between the ‘full’ and ‘reduced’ model (Love et al., 2014).

	Set A		Set E		GLM		
	KO RUN vs KO CTL		KO RUN vs WT RUN				
Gene name	log2Fold change	P-value	log2Fold change	P-value	P-value	Gene function correlated to stem cell activation	References
Tfap2c	2.5099	0.0041173	2.1996	0.022623	0.0017784	Promotes hippocampal neurogenesis	Mateus-Pinheiro et al. (2017)
Npw	1.8813	0.0005558	1.0357	0.043069	0.043133	Hippocampal signaling	Chottova Dvorakova (2018)
Nlrc5	1.7969	0.0000344	0.91737	0.045703	0.0014689	Negative ROS regulator	Li et al. (2021a)
Mki67	1.5041	0.0000001	1.487	2.12E-05	0.00055784	Cell cycle regulator	Scholzen and Gerdes (2000)
Lepr	1.4658	0.0000007	0.80141	0.001078	0.0041875	Synaptic plasticity regulator	Dhar et al. (2014)
Clcn1	1.3064	0.0079431	0.74279	0.0053492	0.0098998	Synaptic regulator	Imbrici et al. (2015)
Cyp11a1	1.1998	0.0001284	0.54095	0.023468	0.010046	Hippocampus response to injury	Chia et al. (2008)
Top2a	1.1833	0.00011062	1.4402	2.84E-05	0.0081051	Cell cycle regulator	Gómez-Herreros et al. (2014)
Eomes	1.1415	0.008134	2.38	1.88E-05	0.00023908	Hippocampal progenitor cell marker	Nelson et al. (2020)
Zic4	1.099	0.01369	0.91747	0.042865	0.04725	Activator of stem cell proliferation	Blank et al. (2011)
Pole	1.0115	0.0000071	0.34811	0.039086	0.011691	Cell cycle regulator	Hogg and Johansson (2012)
Insm2	0.99643	0.0470330	1.1039	0.044601	0.02797	Glycolitic pathway activator	Wang et al. (2018)
Mocos	0.94776	0.0000387	0.3783	0.020257	0.015645	Negative ROS regulator	Féron et al. (2016)
Unc13d	0.89326	0.0000370	0.46491	0.024812	0.025937	Synaptic plasticity regulator	Dittman (2019)
Slc18a2	0.88805	0.0012173	0.86778	0.0011748	0.016933	Synaptic plasticity regulator	Stefanovic et al. (2016)
Prc1	0.87277	0.01267	1.1565	0.0027045	0.0083644	Cell cycle regulator	Mollinari et al. (2002)
Cenpf	0.82583	0.00035633	0.8323	0.00013571	0.0014211	Synaptic plasticity regulator	Tanaka et al. (2012)
Gstm2	0.77843	0.0048713	0.81297	0.0047004	0.021936	Negative ROS regulator	Segura-Aguilar et al. (2014)
Dpf3	0.44617	0.000012	0.28425	0.0014714	0.0006557	Promotes stem cell proliferation	Lessard et al. (2007)
Trp73	0.41055	0.04942	0.31332	0.027558	0.018847	Promotes stem cell proliferation	Talos et al. (2010)
Ncapd2	0.38713	0.0001085	0.16293	0.04469	0.023229	Promotes cell proliferation	He et al. (2023)
Lpin2	0.37591	8.83E-11	0.17873	0.015751	0.00075486	Fatty acid metabolism regulator	Chen et al. (2015)
Dbn1	0.37119	0.000000031	0.14431	0.010052	0.0081834	Synaptic plasticity regulator	Jung et al. (2015), Aoki et al. (2005)
Grm1	0.30756	0.00023736	0.20714	0.0025639	0.0018544	Synaptic plasticity regulator	Baskys et al. (2005)
Cog1	0.26607	0.00024705	0.099728	0.047439	0.0038386	Regulator of neuronal vesicles	Climer et al. (2018)
Zfp143	0.26463	0.0049829	0.18189	0.043793	0.0011626	Regulator of stemness	Choi et al. (2012)
Gabbr2	0.22451	0.000032	0.13118	0.024323	0.0091136	Synaptic plasticity regulator	Heaney and Kinney (2016)
Glrb	−0.14419	0.036066	−0.14323	0.0083886	0.0010861	Synaptic plasticity regulator	Lee et al. (2017)
Cnbp	−0.22151	0.0021957	−0.14838	0.0061765	0.031318	Proliferation regulator	Calcaterra et al. (2010)
Nrxn1	−0.23221	0.00000080	−0.10842	0.013282	0.013987	Synaptic plasticity regulator	Luo et al. (2021)
Mzt1	−0.2335	0.00020106	−0.15501	0.014654	2.71E-06	Cell division regulator	Batzenschlager et al. (2014)
Purb	−0.24099	0.00065256	−0.1747	0.00039673	2.41E-05	Exercise-dependent synaptic plasticity regulator	Chen et al. (2007)
Fgf12	−0.25472	0.00087818	−0.15989	0.01975	0.032654	Synaptic plasticity regulator	Zhang et al. (2012)
Pbx1	−0.26346	0.0000677	−0.1229	0.016314	0.043764	Stem cell regulator	Hau et al. (2021)
Nudt3	−0.28487	0.0015394	−0.17299	0.032189	0.013985	Involved in insulin signaling	Williams et al. (2015)
Pou3f3	−0.31284	0.0000008	−0.17004	0.038589	0.00028684	Stem cell regulator; favors quiescence	Castro et al. (2006)
Galk1	−0.34371	0.0032957	−0.26377	0.041561	0.0028659	Glycolysis regulator	Oh et al. (2020)
Crim1	−0.3589	0.0016505	−0.24056	0.013359	0.00066872	Involved in neural development	Ponferrada et al. (2012)
Rgs14	−0.38356	0.0000044	−0.22733	0.0043943	8.33E-05	Synaptic plasticity negative regulator	Evans et al. (2018)
Grm3	−0.50434	0.00086289	−0.15248	0.045464	0.020496	Synaptic plasticity regulator, Neurotransmitter receptor	Dogra et al. (2021)
Ttc9	−0.5047	0.0000805	−0.33005	0.01287	0.002817	Cell migration ?	Cao et al. (2008)
Klhl4	−0.50857	0.0017137	−0.31945	0.02117	0.0069358	Cell cycle regulator	Choi et al. (2020)
Gabra3	−0.595	0.0017071	−0.2893	0.041659	0.015167	Synaptic plasticity regulator, Neurotransmitter receptor	Wagner et al. (2021)
Ramp3	−0.75442	0.0010749	−0.60432	0.018916	0.015335	Pro-differentiative	Li et al. (2021b)
Sp8	−1.0514	0.0048701	−0.88825	0.022895	0.012781	Marker of quiescent stem cells	Zhang et al. (2016)
Chrm5	−1.3817	0.010806	−1.0349	0.025201	0.026236	Synaptic plasticity regulator, Neurotransmitter receptor	Sakata and Overacre (2017)
Nov	−1.6364	0.034359	−0.85695	0.0011469	0.016149	Inhibitor of progenitor cell proliferation	Le Dréau et al. (2010)

The Fisher’s Exact test was used to determine the statistical significance of the overlap between differentially expressed genes from different data sets (e.g., Set B genes vs the CellAge Senescence database; Supplementary Table S3), or the probability that a Gene Ontology functional class is enriched in DE genes (Figure 2, Supplementary Table S2, Supplementary Table S3, Supplementary Table S7, Supplementary Table S8). Moreover, the statistical significance of stem cells number in immunohistochemistry data was evaluated by two-way Analysis of Variance (ANOVA) to test the main effects of both genotype and running, while individual between-group comparisons to evaluate simple effects were carried out by Fisher’s PLSD ANOVA *post hoc* test (Figures 5C, D).

Real-time PCR data, after performing Levene’s and Bartlett’s tests to assess the homogeneity of variance, were analyzed by non-parametric tests, being fold-change data; Kruskal-Wallis test was used for statistical analysis when the main effects of both genotype and running were evaluated (Figure 4B; Figure 5E); individual between-group comparisons (i.e., simple effects in Set B, Set A, Set D, Set E comparisons) were analyzed by the Mann-Whitney U *post hoc* test (Figures 4A, B; Figure 5E).

Kruskal-Wallis and Mann-Whitney U tests were carried out using the Stat View 5.1 software (SAS Institute, Cary, NC, USA), Levene’s and Bartlett’s tests by XLSTAT (Addinsoft, Paris, France). Differences were considered statistically significant at *p*-value <0.05. Real-time PCR and immunohistochemistry data were expressed as mean ± SEM.

## Results and discussion

Our aims were i) to identify genes differentially regulated by p16 knockout, relative to wild-type, i.e., genes representing the genetic signature of p16 knockout in the aged dentate gyrus; ii) to identify genes responsible for, or correlated with, the activation of stem cell proliferation exerted by running in the dentate gyrus of aged p16 knockout mice, as previously observed (Micheli et al., 2019); iii) to investigate the effect of hyperstimulation of stem cells through a repeated stimulus and the correlation with the expression of the stem cell-specific genes identified in this study. This would allow to define the role of p16 in the maintenance of the stem cell pool.

### Global differences in gene expression induced by running in the dentate gyrus of the aged p16 knockout model

By RNA sequencing we assessed the transcriptomic profiles of the dentate gyrus isolated from the same mouse model used by Micheli et al., 2019, i.e., one-year-old p16 wild-type or *p16* knockout mice, submitted to physical exercise or sedentary. The wild-type and knockout mice had free access to a running wheel (called here p16 WT RUN and p16 KO RUN) for 12 days, following a protocol used previously (Farioli-Vecchioli et al., 2014; see protocol scheme and timeline in Figure 1A).

At the end of the 12-day exercise regimen, the dentate gyrus from p16 WT RUN and p16 KO RUN mice, as well as the dentate gyrus from control sedentary p16 wild-type (p16 WT CTL) and p16 knockout animals (p16 KO CTL), were isolated according to a described procedure (Hagihara et al., 2009), and processed for transcriptome analysis.

As a preliminary analysis, we examined the global gene expression to identify the differentially expressed (DE) genes in different pairwise comparisons, performed by the DESeq2 software using the Wald test (Love et al., 2014), with the significance threshold of the *p*-value of differential gene expression (log_2_ fold change) set to *p*-value <0.05.

The pairwise comparisons taken into consideration here were defined as Set A (p16 KO RUN vs p16 KO CTL, 3,272 genes), Set B (p16 KO CTL vs p16 WT CTL, 1,230 genes), Set C (p16 WT RUN vs p16 WT CTL, 762 genes), Set D (p16 KO RUN vs p16 WT CTL, 1,475 genes) and Set E (p16 KO RUN vs p16 WT RUN, 494 genes); see the Venn diagram, Figure 1B, showing the gene numbers belonging to these pairwise comparisons, with *p*-value <0.05 as well as the intersection of the differentially expressed gene sequences in sets A-E.

Set D, Set A and Set E comparisons were used to select genes with pattern of expression changes comparable to the proliferative changes exerted by running in p16 knockout stem cells, as observed in Micheli et al. (2019), whereas Set B comparison was taken into consideration to identify the p16 knockout signature (see below).

In global terms, Set A (KO RUN vs KO CTL, 3,272 genes) is the most abundant in DE genes, possibly because it incorporates a double differential regulatory effect, that of running in KO mice relative to wild-type control (Set D), and the effect of KO control relative to wild-type control (Set B). Notably, the knockout of p16, compared to wild-type control, has in itself a deregulatory effect on many genes (Set B, 1,230 genes). Whereas Set E is the least abundant set of DE genes, probably because there is a limited number of genes differentially regulated by running in KO mice, relative to the effect of running in wild-type mice - an effect not active in wild-type stem cells. Thus, Set E should include, similarly to Set A and Set D, stem cell-specific genes regulated by KO and by running.

### Genetic signature of the p16 knockout in the aged dentate gyrus (set B comparison)

The first aim of this report was, as mentioned above, to identify genes differentially regulated in p16 knockout versus wild-type, thus, genes representing the genetic signature of p16 knockout in the aged dentate gyrus. We analyzed the DE genes of the Set B comparison (p16 KO CTL vs p16 WT CTL) presenting significance threshold of the differential gene expression at *p*-value <0.05. This analysis yielded 1,230 genes that resulted differentially induced or reduced by p16 knockout (listed in Supplementary Table S1).

A selection of the top significant DE genes of Set B with highest significance (*p*-value **≤** 0.0001, 27 genes) and the corresponding Gene Ontology biological processes (GOBP) enriched in that gene list, are shown in Figure 2. Among the genes upregulated by p16 knockout, we find genes that are upregulated in neurodegeneration and inflammation, such as the proapoptotic serine/threonine kinase *Eif2ak2* (Tible et al., 2019), or the C-C motif chemokine receptor 1 (*Ccr1*) linked to neuro-inflammation (Ciechanowska et al., 2020), or the neuroprotective molecule *Cd59a* that protects against cell injury after brain damage (Stahel et al., 2009; Britschgi et al., 2012). Interestingly, the increased expression in p16 knockout of the proapoptotic gene *Eif2ak2* is consistent with the five-fold increase of apoptosis observed in the p16 knockout dentate gyrus (Micheli et al., 2019). Moreover, we observe an increase of lipoprotein Lipase (*Lpl*) expression, which may be associated with the repair of cellular damage (Loving and Bruce, 2020). This may indicate that the ablation of p16 leads to an inflammatory and dismetabolic state, without evident effect on neurogenesis, as no change is observed in stem and progenitor cells or dentate gyrus neurons (Micheli et al., 2019). Other genes upregulated in Set B are involved in cell signaling, such as *Magt1* that activates the Mapk pathway (Bi et al., 2021), and *Homer2*, involved in glutamate signaling (Smothers et al., 2016), or genes activated in conditions involving neuroplasticity, namely, *Slc16A1*, the most abundant lactate transporter in the central nervous system, involved in activation of cellular brain metabolism and pH control (Halliday et al., 2019); or *NTSr2* that regulates NOS expression and activity at the synapse (Lores-Arnaiz et al., 2017); or also *Rhoq*, which plays an important role in axon elongation (Koinuma et al., 2021).

All this suggests that the ablation of p16 generates in the stem and progenitor cells a “priming” condition favorable to react to or to be activated by a stimulus. The set of genes downregulated in Set B includes the tumor suppressor *Trabd2b* (TraB domain containing 2B), which acts in glioma and osteosarcoma cells by inhibiting the Wnt signaling pathway, with consequent reduction in proliferation, colony formation ability and invasion of glioma and osteosarcoma cell lines (Li et al., 2014). This, although not obviously linked to an evident phenotype in the p16 KO dentate gyrus, is in line with the known pro-tumorigenic effect of p16 ablation (Sharpless et al., 2001; LaPak and Burd, 2014) and with the prompt proliferative response to stimulus.

The biological processes significantly enriched in the top DE genes of Set B (as judged by *p*-value), shown in Figure 2, are associated with either cellular signaling (GOBP_Cell cell signaling: *Ccr1*, *Slc16a1, Trabd2b*; GOBP_Negative regulation of signaling: T*rabd2b*, *Homer2*), or with different types of cellular responses, e.g., GOBP_Cellular response to endogenous stimulus, and GOBP_Chemokine production: *Lpl* and *Eif2ak2*, consistently with a role in neurodegeneration and neuroinflammation. Other gene-enriched processes are GOBP__Biosynthetic process: *Magt1*, and GOBP_cell projection assembly: *Rhoq*, matching with their role in development and axon elongation (Figure 2). Moreover, a broad category is the GOBP_Response to oxygen-containing compound, comprising the upregulated genes *Lpl*, *Homer2* and *Rhoq*, which may underlie an availability to respond to stimulus.

An expanded analysis of the Gene Ontology Biological Processes, Cellular Components (GOCC) and Molecular Functions (GOMF), enriched in the Set B genes with log2 fold change DE *p*-value <0.01 (310 genes), is shown in Supplementary Table S2. Among the Biological processes enriched with greater number of differentially expressed genes there are genes related to cell cycle (GOBP_Cell cycle, 28 genes, *p*-value = 0.015), to nitric oxide generation (GOBP_Organonitrogen_compound_biosynthetic_process, 27 genes, *p*-value = 0.015) or to neurogenesis (GOBP_Neurogenesis, 22 genes, *p*-value = 0.013) and, as mentioned above regarding Figure 2, GOBP_ Cell_cell_signaling, 22 genes, *p*-value = 0.0135, and GOBP_Response to oxygen containing compound, 22 genes, *p*-value = 0.0133.

### Involvement of p16 knockout signature genes in the process of senescence

It is known that senescence is a critical process in which p16 plays a role as inducer (Rayess et al., 2012); however, this did not emerge through the GO enrichment analysis of Set B. Thus, to identify genes potentially regulated by senescence and associated with Set B genes, we compared the whole Set B (1,230 genes with log2 fold change DE *p*-value <0.05) with the senescence signature from the CellAge database (https://genomics.senescence.info/cells/signatures.php?) comprising 1,259 genes, and found that 91 genes are in common, with significant enrichment of Set B in the CellAge database of senescence genes (Fisher’s exact test, *p* < 0.0001). Of these, we selected the Set B genes whose expression is counter-regulated (43), with respect to the CellAge Senescence genes (Supplementary Table S3 **)**, as we reasoned that the p16 ablation should contrast the changes in age-induced senescence genes (Molofsky et al., 2005; Micheli et al., 2019). We analyzed the GO biological processes enriched in the counter-regulated genes and found that the most significant process involves neuron death (GOBP_Neuron_death, *p*-value = 0.000002, *Gba*, *Btg2*, *Tnfrsf1b*, *Gpnmb*, *Fas*, *Ncstn*) (Supplementary Table S3). In fact, the Tnf receptor *Tnfrsf1b* induces apoptosis (Grabinger et al., 2017) and its expression is upregulated in Set B comparison, while *Ncstn* (Nicastrin) and *Btg2*, both of which inhibit apoptosis (Corrente et al., 2002; Micheli et al., 2015; Wang et al., 2020), are downregulated in Set B. The opposite regulation occurs during senescence, where *Btg2* acts as an important inhibitor of neural proliferation and inducer of terminal differentiation (Farioli-Vecchioli et al., 2009; 2008). Moreover, *Fas* (cell surface death receptor), which is activated in senescent cells and is downregulated in Set B, exerts neuroprotective activities in neural progenitor cells (Knight et al., 2010). All this would be consistent with the increase of apoptosis observed in p16 knockout and with a counter-regulation of these genes occurring during senescence.

More generally, the p16 knockout causes disinhibition of cell cycle, which is known to be associated with an increase in apoptosis (Pucci et al., 2000), and this may lead to a conflict with the aging condition that would instead make cells prone to quiescence and senescence.

### Identification of genes correlated to the activation of the proliferation of stem cells exerted by running in p16 knockout dentate gyrus

The second aim of this study is the identification of genes correlated to the phenotype of proliferative activation of aged stem cells due to running, occurring in p16 knockout but not in p16 wild-type dentate gyrus, as described by Micheli et al. (2019). This response to running implies that the deletion of p16 allows stem cells to be in a more permissive condition to respond to a neurogenic stimulus (Micheli et al., 2019).

**FIGURE 3:**
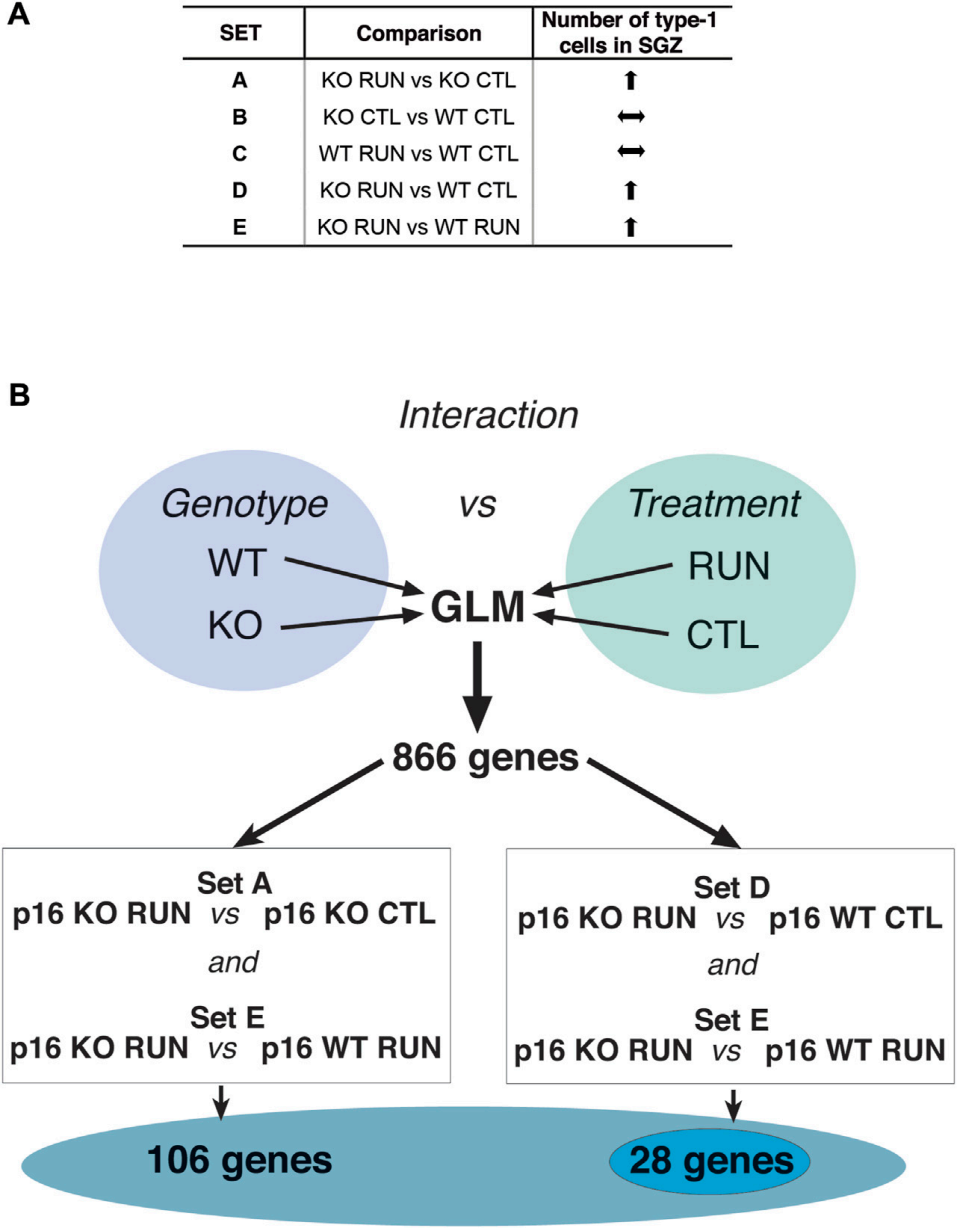
Flow chart of data analyses to identify genes correlated to stem cell activation by running in p16 knockout dentate gyrus. **(A)** Scheme of the pattern of proliferative response of stem cells in p16 knockout dentate gyrus SGZ by running, in the different pairwise comparisons between groups. This pattern is obtained from the data presented in Micheli et al. (2019). **(B)** To identify genes fitting with the pattern of proliferative activation of stem cells induced by running in p16 knockout dentate gyrus, relative to all other groups (p16 WT CTL, p16 WT RUN and p16 KO CTL), we performed an analysis by the General Linear Model (GLM), as part of the DESeq2 software package. After calculating by DESeq2 the significance of different pairwise comparisons of differentially expressed (DE) genes, through the Wald test (Love et al., 2014), the GLM analysis was used to identify the genes showing interaction between genotype and treatment by means of the Likelihood Ratio Test between a “full” model, comprising all factors (genotype and treatment) including their interaction, and a “reduced” model including all factors except their interaction. The DE genes with significant differences (i.e., interaction) in the effect of treatment over genotype, had *p*-value <0.05 in the difference between the ‘full’ and ‘reduced’ model. We found 866 genes with significant interaction between genotype and running. Among these genes we further selected those with significant expression difference within both the Set A (p16 KO RUN vs p16 KO CTL) and the Set E (p16 KO RUN vs p16 WT RUN) comparisons, obtaining in this way 106 genes, or within both the Set D (p16 KO RUN vs p16 WT CTL) and the Set E (p16 KO RUN vs p16 WT RUN) comparisons, thus identifying 29 genes. Both gene selections, named SetA_SetE_GLM and SetD_SetE_GLM, respectively, included genes showing the highest differential expression between the p16 KO RUN group and all other three groups, similar to the pattern of proliferative activation of stem cells elicited by running in p16 KO mice. Their function appears related to stem cell activation; see text. 28 out of 29 genes of SetD_SetE_GLM resulted comprised within the 106 genes of SetA_SetE_GLM, indicating that the two ensembles overlap.

Thus, we sought genes whose transcriptomic profile showed a pattern similar to the phenotypic response of p16 knockout stem cells, i.e., a strong activation by running, relative to all other groups (p16 WT CTL, p16 WT RUN and p16 KO CTL). See in Figure 3A the outline of the phenotypic (proliferative) response of stem cells to running, according to the data published by Micheli et al. (2019).

#### Evaluation of genotype vs treatment interaction by generalized linear model analysis

First, we analyzed the whole transcriptome using the Generalized Linear Model (GLM, see Materials and Methods), which allows to identify the genes presenting interaction between genotype and treatment (running), i.e., the genes that show differences of expression in the effect of treatment depending on genotype. In this way we identified 866 genes with significant interaction between genotype and treatment (*p*-value <0.05, Likelihood ratio test LRT; Supplementary Table S4). We considered this as a preliminary selection of genes differentially regulated by running in the p16 knockout genotype, relative to all other groups.

#### Selection of DE genes common to set D and set E pairwise comparisons (Set D_Set E_GLM gene selection)

To further select genes activated by running exclusively in p16 knockout dentate gyrus group, we searched among the 866 GLM genes, for those whose expression was significantly changed by running in p16 knockout mice, relative to both p16 wild-type sedentary mice (pairwise comparison Set D: p16 KO RUN vs p16 WT CTL, *p* < 0.05), and to p16 wild-type running mice (pairwise comparison Set E: p16 KO RUN vs p16 WT RUN, *p*-value <0.05) (see scheme in Figure 3B).

Thus, we assumed that the genes belonging to this selection (named Set D_Set E_GLM selection) were correlated to the phenotypic response of stem cells to running, and specifically focused on the difference induced by running in p16 knockout versus p16 wild-type, either sedentary or running (Table 1; full display in S5, 29 genes).

Representative genes of Set D_Set E_GLM selection, shown in Table 1, are some regulators of synaptic plasticity and function: *Lepr* (Dhar et al., 2014), *Nlrc5* (Li L. et al., 2021), *Slc18a2* (Stefanovic et al., 2016), *Unc13d* (Dittman, 2019), *Ttc9* (Guan et al., 2020) and *Cenpf* (Tanaka et al., 2012); moreover, the gene *Zic4* involved in neural cell proliferation (Blank et al., 2011), the negative regulators of reactive oxygen species (ROS) levels *Nlrc5*, *Gstm2* (Segura-Aguilar et al., 2014) and *Mocos* (Féron et al., 2016); the regulator of fatty acid metabolism *Lpin2* and the prodifferentiative gene *Ramp3* (downregulated in Set D; Li C. et al., 2021).

#### Selection of DE genes common to set A and set E pairwise comparisons (Set A_Set E_GLM gene selection)

A complementary approach that we followed to pinpoint genes correlated to the activation of stem cells by running was to search among the 866 GLM genes for those whose expression was significantly changed by running in p16 knockout mice, relative to both p16 knockout sedentary mice (Set A, pairwise comparison: p16 KO RUN vs p16 KO CTL, *p* < 0.05), and to p16 wild-type running mice (Set E, i.e., p16 KO RUN vs p16 WT RUN, *p* < 0.05); the whole procedure is outlined in Figure 3B. This latter approach (named Set A_Set E_GLM selection; see representative genes in Table 2, and Supplementary Table S6 for full display) yielded a greater number of genes (106) than the Set D_Set E_GLM selection, since the Set D comparison is more restricted than that of Set A, because it accounts for significant differences of expression in both genotype and treatment factors. Clearly, the Set D_Set E_GLM selection and the Set A_Set E_GLM selection account mainly for the expression changes occurring within the pairwise comparisons of Set D and Set A, respectively (both filtered also by the Set E comparison). Of note, 28 out of 29 genes of Set D_Set E_GLM were comprised within the set of 106 genes (see below).

The 106 genes of Set A_Set E_GLM selection include regulators of stem and progenitor cell proliferation: *Zic4*, *Prc1*, *Dpf3*, *Trp73*, *Klhl4* (Lessard et al., 2007; Talos et al., 2010; Blank et al., 2011; She et al., 2019; Choi et al., 2020), and several regulators of synaptic plasticity and neurotransmitter release, either upregulated: *Lepr, Slc18a2, Unc13d, Dbn1, Grm1, Gabbr2,* or downregulated: *Glrb, Nrxn1, Fgf12, Rgs14, Grm3, Gabra3, Chrm5* (Dhar et al., 2014 [*Lepr*]; Stefanovic et al., 2016 [*Slc18a2*]; Dittman, 2019 [*Unc13d*]; Jung et al., 2015 [*Dbn1*]; Bagot et al., 2012 [*Grm1*]; Heaney and Kinney, 2016 [*Gabbr2*]; Lee et al., 2017 [*Glrb*]; Luo et al., 2021 [*Nrxn1*]; Zhang et al., 2012 [*Fgf12*]; Evans et al., 2018 [*Rgs14*]; Dogra et al., 2021 [*Grm3*]; Wagner et al., 2021 [*Gabra3*]; Sakata and Overacre (2017) [*Chrm5*]). Moreover, the 106 genes of Set A_Set E_GLM include also other upregulated genes that may play a key role in the activation of stem cells, i.e., the regulator of the glycolitic pathway *Insm2* (Wang et al., 2018), negative regulators of ROS level, present also in Set D_Set E_GLM selection: *Nlrc5*, *Gstm2*, *Mocos* (Segura-Aguilar et al., 2014; Féron et al., 2016; Li L. et al., 2021), and the controller of fatty acid metabolism *Lpin2* (Chen et al., 2015) (see Table 2).

Several genes of both Set D_Set E_GLM and Set A_Set E_GLM selections have also an impact on cell cycle, namely, *Rgs14* (Martin-McCaffrey et al., 2005), *Cenpf* (Zhou et al., 2019), *Prc1* (Mollinari et al., 2002), *Mki67* (Scholzen and Gerdes, 2000), *Top2a* (Gómez-Herreros et al., 2014), *Pole* (Hogg and Johansson, 2012), as would be expected in a process of stem cells reactivation (Table 1). The function of the SetA_Set E_GLM genes cited above is further discussed in relation to their up-or downregulation in the section below: “Function of genes related to stem cell activation by running in p16 knockout dentate gyrus, differentially expressed in Set A_Set E_GLM selection”.

Overall, Set A_Set E_GLM gene selection appears correlated to the phenotype of stem cells activation (i.e., significant expression changes occurring only in the p16 KO RUN group relative to the other three groups p16 WT RUN, p16 WT CTL, p16 KO CTL, as depicted in Figure 3A scheme). In fact, the large majority of Set A_Set E_GLM genes display significant differences between KO RUN and all other groups, which instead do not show significant differences amongst them.

Interestingly, 22 genes out of the 106 of Set A_Set E_GLM belong to the p16 KO gene signature included in Set B (evidenced in red in Table 2). Despite Set B stands clearly as a gene ensemble different from Set A_Set E_GLM, these 22 genes (out of a total of 1,230 genes of Set B) may be considered relevant to the phenotype of stem cells activation because they show significant changes of expression between p16 KO RUN and p16 WT RUN (Set E), and are in line with the previously mentioned idea that the ablation of p16 produces in the stem and progenitor cells a “priming” condition favorable to being activated by a stimulus.

Moreover, the fact that virtually all genes of Set D_Set E_GLM selection were included among the 106 genes of Set A_Set E_GLM, indicates that the first gene ensemble is a subset of the second.

### Validation by real-time PCR of DE genes

**FIGURE 4:**
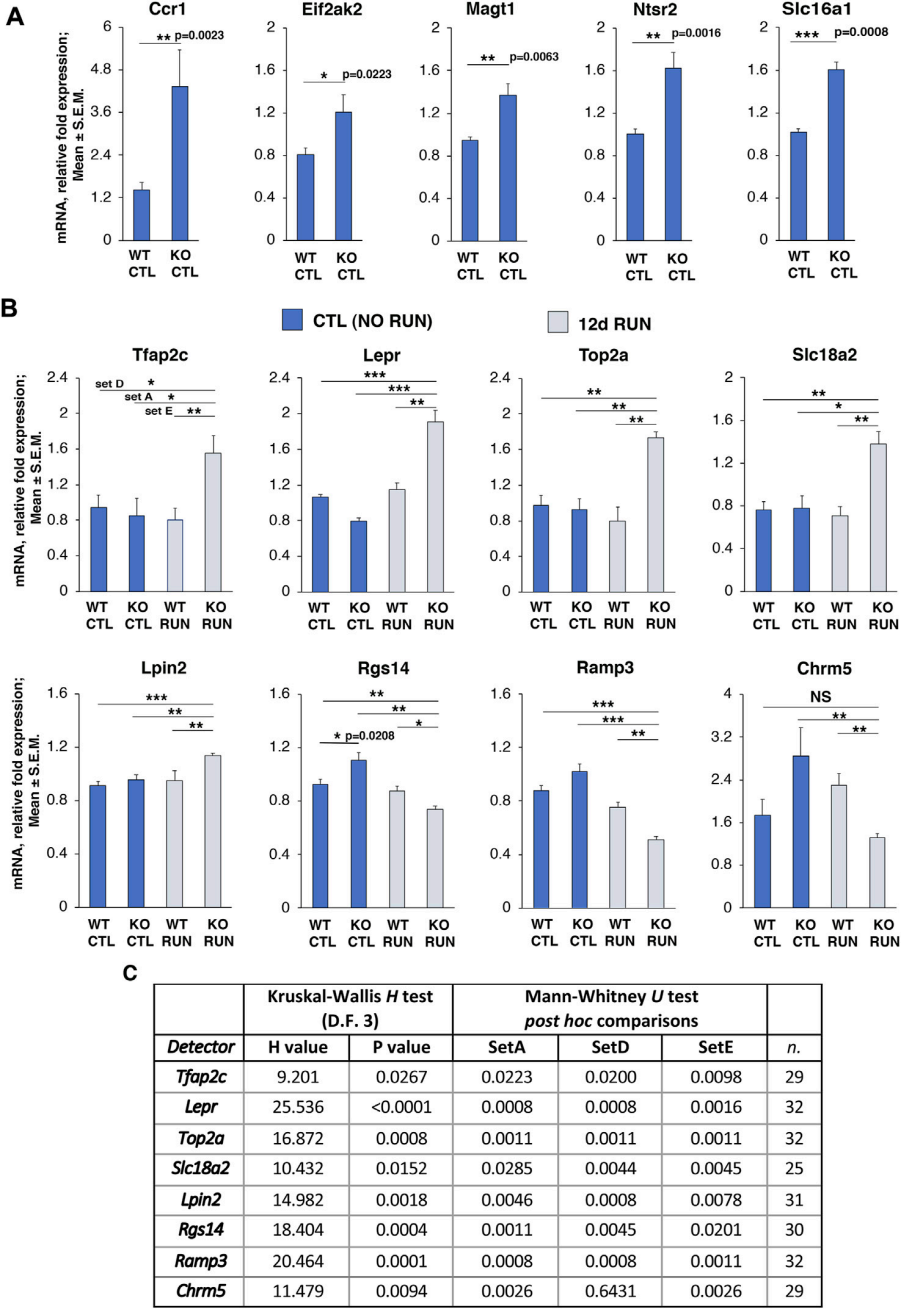
Validation by real-time PCR in dentate gyrus of genes differentially regulated by p16 KO and by running in Set B and in Set D_Set E_GLM as well as in Set A_Set E_GLM selection. **(A)** Validation by real-time PCR, in the dentate gyrus isolated from one-year-old mice, of six genes up-regulated in Set B comparison, i.e., p16 KO CTL vs p16 WT CTL, with *p* **≤** 0.05 (*Ccr1*, *Eif2ak2*, *Magt1*, *Ntsr2*, and *Slc16a1*), and **(B)** *Rgs14* (also regulated in other Sets) with *p* < 0.05. The average mRNA fold expression ±standard error of the mean (mean ± SEM) is plotted. Mean ± SEM fold changes are from two independent experiments. TBP was used to normalize data. *p < 0.05, **p < 0.01, ***p < 0.001, Mann-Whitney U *post hoc* test was used to detect simple effects; Set B data of Rgs14 were previously analyzed for main effects by Kruskal-Wallis test, since other Sets comparisons were analyzed as well (*p* < 0.0004, see Table at panel **(C)**. **(B)** Validation by real-time PCR of eight genes differentially regulated in Set A, Set E and Set D with statistical significance (*p*-value <0.05); upregulated genes: *Tfap2c*, *Lepr*, *Top2a*, *Slc18a2*, *Lpin2,* downregulated genes*: Rgs14, Ramp3*, *Chrm5*. The figure shows the mean mRNA fold expression changes ±SEM from two independent experiments. Number of biological replicates per group = 4. TBP was used to normalize data. The statistical analysis of real-time PCR data was performed by non-parametric Kruskal-Wallis test to detect main effects, followed by Mann-Whitney U *post hoc* test for single Set comparison and is shown in detail in the Table in **(C)**. *p < 0.05, **p < 0.01, ***p < 0.001, Mann-Whitney U *post hoc* test. **(C)** Full analysis by Kruskal-Wallis and Mann-Whitney U test of real-time PCR data of the eight genes differentially regulated in Set A, Set E and Set D, shown in panel **(B)**: shown are H values and *p*-values of Kruskal-Wallis test, and the Mann-Whitney U *post hoc* test *p*-values for each Set of pairwise comparison. n = total number of data analyzed for the four data groups (WT CTL and WT RUN, KO CTL and KO RUN).

The expression changes observed by RNA-seq of five top genes of Set B with statistical significance *p* **≤** 0.0001, *Ccr1*, *Eif2ak2*, *Magt1*, *Ntsr2*, and *Slc16a1* (see Figure 2), and of the gene *Rgs14* with Set B statistical significance *p*-value <0.05, were analyzed by real-time PCR in the dentate gyri isolated from one-year-old mice belonging to the groups analyzed. The real-time PCR expression values of these genes confirmed that they were significantly increased in Set B, as originally observed by RNA seq (*Ccr1 p* = 0.0023, *Eif2ak2 p* = 0.0022, *Magt1 p* = 0.006, *Ntsr2 p* = 0.001, *Slc16a1* and *Rgs14 p* = 0.002, Mann-Whitney U test, after analysis of main effects by Kruskal-Wallis test; see graphs in Figures 4A, B).

We also assayed for validation by real time PCR the fold changes observed by RNA seq in 8 genes differentially expressed in both Set D_Set E_GLM and Set A_Set E_GLM selections, namely, *Tfap2c*, *Lepr*, *Top2a*, *Slc18a2*, *Lpin2, Rgs14, Ramp3*, and *Chrm5*.

We found that the expression values of these genes showed significant changes in Set A, Set D and in Set E that corresponded to those observed by RNA seq (*Tfap2c*, *Lepr*, *Top2a*, *Slc18a2*, *Lpin2, Rgs14, Ramp3*, *Chrm5:* set A [p16 KO RUN vs p16 KO CTL]: *p* ≤ 0.02, set D [p16 KO RUN vs p16 WT CTL]: *p* ≤ 0.02, set E [p16 KO RUN vs p16 WT RUN]: *p* ≤ 0.02, Mann-Whitney U *post hoc* test, after analysis of main effects by Kruskal-Wallis test). Of note, Chrm5 expression did not change significantly in Set D, as determined either by RNA seq, or, as expected, by real-time PCR (*p* = 0.643). See Figure 4B, and Figure 4C for the full statistical analysis, with *p*-values and n for each gene.

### Enrichment analysis of DE genes regulated by running and putatively related to stem cell activation in the dentate gyrus of p16 knockout mice

By evaluating the Gene Ontology (GO) databases, we then aimed to pinpoint the biological processes that are significantly enriched in the genes putatively related to stem cell activation, belonging to Set A_Set E_GLM or to Set D_Set E_GLM selection.

In particular, the 106 genes differentially expressed in Set A_Set E_GLM are significantly enriched in biological processes that appear representative of the activation of stem cells exerted by running in p16 knockout dentate gyrus: a selection of GO Biological Process is shown in Table 3.

**TABLE 3:** Most representative biological processes (GOBP terms) enriched in the 106 DE genes of Set A_Set E_GLM selection, i.e., the full set of genes activated by running in p16 knockout dentate gyrus with an expression pattern fitting the proliferative activation of stem cells. This table shows the list of the most representative Gene Ontology biological processes (GOBP) from the human database, whose enrichment in the 106 genes of SetA_SetE_GLM ensemble results statistically significant with q-value <0.05. These genes have been previously selected as described in Figure 3. Thus, the pattern of differential expression of all SetA_SetE_GLM genes in the different groups reproduces, though with broader extent than SetD_SetE_GLM, the pattern of the proliferative activation by running of p16 KO stem cells (see also Table 2). The enrichment *p*-value (<0.05) of SetA_SetE_GLM genes shown here in each functional GO class has been calculated using the Fisher’s Exact test, and the provided FDR q-value is the *p*-value adjusted for multiple testing with the Benjamini-Hochberg procedure to control the false discovery rate. GO term ID and Gene Set Name columns contain the identifier and the name of the GO group. K is the list size: total number of genes belonging to each GO process. k: number of DE genes referred to the genes of Set A_Set E_GLM selection, mapping to that GO group. The DE genes associated to each GO class are listed on the last right column. (See in Supplementary Table S7 the full list of GO categories, GOBP, GOCC and GOMC, i.e., Biological Processes, Cellular Components and Molecular Functions, respectively).

GO term ID	Gene set name	# Genes in gene set (K)	# Genes in overlap (k)	*p*-value	FDR q-value	Enriched DE genes
GO:0007267	GOBP_CELL_CELL_SIGNALING	1,672	21	7.350 E−10	0.0000055	Rgs14 Fgf12 Grm3 Gabbr2 Xiap Gnai1 Chrm5 Slc18A2
						Glrb Grm1 Nrxn1 Dbn1 Gabra3 Cgas Selenot Ccn3
						Pcsk5 Tfap2C Scel Tspan12 Nudt3
GO:0044092	GOBP_NEGATIVE_REGULATION_OF_MOLECULAR_FUNCTION	1,167	17	4.360 E−09	0.0000163	Rgs14 Fgf12 Grm3 Gabbr2 Xiap Ptpa Prkar1A Gstm2
						Nucks1 Ramp3 Ubqln1 Lepr Crim1 Set Eomes Rlim Nlrc5
GO:0099536	GOBP_SYNAPTIC_SIGNALING	712	12	0.0000002	0.0005340	Rgs14 Fgf12 Grm3 Gabbr2 Gnai1 Chrm5 Slc18A2 Glrb
						Grm1 Nrxn1 Dbn1 Gabra3
GO:0000278	GOBP_MITOTIC_CELL_CYCLE	1,032	13	0.0000017	0.0032000	Rgs14 Gnai1 Ptpa Pole Ush1C Knstrn Prc1 Mzt1 Cenpf
						Ncapd2 Mki67 Pbx1 Asah2
GO:0022402	GOBP_CELL_CYCLE_PROCESS	1,415	15	0.0000022	0.0032500	Rgs14 Gnai1 Ptpa Prkar1A Pole Ush1C Knstrn Prc1 Mzt1
						Cenpf Ncapd2 Mki67 Pbx1 Top2A Insm2
GO:0007049	GOBP_CELL_CYCLE	1872	17	0.0000034	0.0042600	Rgs14 Xiap Gnai1 Ptpa Prkar1A Pole Ush1C Knstrn Prc1
						Mzt1 Cenpf Ncapd2 Mki67 Pbx1 Asah2 Top2A Insm2
GO:1901698	GOBP_RESPONSE_TO_NITROGEN_COMPOUND	1,117	13	0.0000041	0.0042600	Gnai1 Chrm5 Slc18A2 Glrb Cgas Prkar1A Gstm2 Nucks1
						Ramp3 Ubqln1 Lpin2 Svip Cyp11A1
GO:0007051	GOBP_SPINDLE_ORGANIZATION	182	6	0.0000065	0.0054300	Rgs14 Gnai1 Ptpa Knstrn Prc1 Mzt1
GO:0007059	GOBP_CHROMOSOME_SEGREGATION	337	7	0.0000221	0.0165000	Rgs14 Knstrn Prc1 Cenpf Ncapd2 Mki67 Top2A
GO:0009719	GOBP_RESPONSE_TO_ENDOGENOUS_STIMULUS	1,624	14	0.0000485	0.0259000	Fgf12 Xiap Gnai1 Chrm5 Prkar1A Gstm2 Nucks1 Ramp3
						Lepr Crim1 Knstrn Lpin2 Cyp11A1
GO:0051276	GOBP_CHROMOSOME_ORGANIZATION	1,244	11	0.0000611	0.0278000	Nucks1 Set Pole Knstrn Prc1 Cenpf Ncapd2 Mki67 Top2A
						Dpf3 Pabpc1L
GO:0007610	GOBP_BEHAVIOR	541	8	0.0000631	0.0278000	Rgs14 Fgf12 Slc18A2 Glrb Grm1 Nrxn1 Lepr Npw
GO:0006518	GOBP_PEPTIDE_METABOLIC_PROCESS	903	10	0.0000851	0.0303000	Selenot Pcsk5 Gstm2 Cnbp Rps27 Rps5 Rps12 Etf1
						Mrps14 Eef1B2
GO:0051301	GOBP_CELL_DIVISION	600	8	0.0001290	0.0357000	Gnai1 Knstrn Prc1 Cenpf Ncapd2 Top2A Pou3F3
GO:0000819	GOBP_SISTER_CHROMATID_SEGREGATION	199	5	0.0001440	0.0383000	Knstrn Prc1 Cenpf Ncapd2 Top2A
GO:0006821	GOBP_CHLORIDE_TRANSPORT	109	4	0.0001620	0.0419000	Glrb Gabra3 Slc26A4 Clcn1
GO:0050877	GOBP_NERVOUS_SYSTEM_PROCESS	1,418	12	0.0002070	0.0458000	Rgs14 Fgf12 Chrm5 Glrb Grm1 Nrxn1 Gabra3 Ccn3
						Ush1C Slc26A4 Clcn1 Col18A1

These processes are involved mainly in the control of synaptic signaling (GOBP_Synaptic_signaling *p*-value = 0.0000002*: Rgs14, Fgf12, Grm3, Gabbr2, Gnai1, Chrm5, Slc18a2, Glrb, Grm1, Nrxn1, Dbn1, Gabra3*), (GOCC_Synapse *p*-value = 0.000003: *Grm1, Gabbr2, Rgs14, Prkar1A, Slc18a2, Gabra3, Glrb, Grm3, Chrm5, Dbn1, Nrxn1, Rps27, Ush1C, Fgf12*) and cell cycle regulation (GOBP_cell_cycle *p*-value = 0.0000034: *Rgs14, Xiap, Gnai1, Ptpa, Prkar1a, Pole, Ush1C, Knstrn, Prc1, Mzt1, Cenpf, Ncapd2, Mki67, Pbx1, Asah2, Top2a, Insm2*); see Table 3, and full list of GO Biological Process, GO Molecular Function, and GO Cellular Component enriched with *p*-value <0.05 in Supplementary Table S7.

On the other hand, the enrichment analysis in the Gene Ontology Biological Process database of Set D_Set E_GLM selection reveals significant enrichment essentially in genes involved in the cell cycle (Table 4; GOBP_ cell cycle process, *p*-value = 0.000011: *Rgs14*, *Cenpf*, *Prc1*, *Mki67*, *Top2a*, *Ush1c*, *Pole*; GOBP_chromosome segregation *p*-value = 0.0000031: *Rgs14*, *Cenpf*, *Prc1*, *Mki67*, *Top2a*; see full list of GO Biological process, GO Cellular Components and GO Molecular functions, enriched with *p*-value <0.05, in Supplementary Table S8).

**TABLE 4:** Biological processes (GOBP terms) enriched in the 29 DE genes of Set D_Set E_GLM selection, i.e., a subset of genes activated by running in p16 knockout dentate gyrus with an expression pattern fitting the proliferative activation of stem cells. List of Gene Ontology Biological Processes (GOBP) from human database, whose enrichment is calculated among the 29 genes of the SetD_SetE_GLM ensemble, i.e., genes whose expression change shows significant interaction of genotype vs treatment (GLM; *p*-value <0.05) and that are also significantly induced or reduced by running in the pairwise comparisons of both Set D (p16 KO RUN vs p16 WT CTL) and Set E (p16 KO RUN vs p16 KO CTL). All SetD_SetE_GLM genes are differentially expressed genes with significant log_2_ fold change (*p*-value <0.05) and their pattern of differential expression appears to reproduce the pattern of proliferative activation of p16 KO stem cells by running (see also Table 1). The enrichment p-values (<0.05) and q-values of these DE genes in each functional class have been calculated using the Fisher’s Exact test (FDR q-value is the *p*-value adjusted for multiple testing with the Benjamini-Hochberg procedure to control the false discovery rate). GO term ID and Gene Set Name columns contain the identifier and the name of the GO group. K is the list size: total number of genes belonging to each GO process. k: number of DE genes referred to the genes of Set D_Set E_GLM selection, mapping to that GO group. The DE genes associated to each GO class are listed on the last right column. (See in Supplementary Table S8 the complete list of GOBP, GOCC, GOMF categories).

GO term ID	Gene set name	# Genes in gene set (K)	# Genes in overlap (k)	*p*-value	FDR q-value	Enriched DE genes
GO:0007059	GOBP_CHROMOSOME_SEGREGATION	350	5	0.0000031	0.024	Rgs14 Cenpf Prc1 Mki67 Top2A
GO:0022402	GOBP_CELL_CYCLE_PROCESS	1,224	7	0.0000113	0.043	Rgs14 Cenpf Prc1 Mki67 Top2A Ush1C Pole
GO:0000278	GOBP_MITOTIC_CELL_CYCLE	900	6	0.0000226	0.058	Rgs14 Cenpf Prc1 Mki67 Ush1C Pole
GO:0044092	GOBP_NEGATIVE_REGULATION_OF_MOLECULAR_FUNCTION	1,172	6	0.0000980	0.156	Cenpf Gstm2 Ramp3 Lepr Nlrc5 Gabbr2
GO:1903047	GOBP_MITOTIC_CELL_CYCLE_PROCESS	745	5	0.0001160	0.156	Cenpf Prc1 Mki67 Ush1C Pole
GO:0007049	GOBP_CELL_CYCLE	1779	7	0.0001220	0.156	Rgs14 Cenpf Prc1 Mki67 Top2A Ush1C Pole

### Function of genes related to stem cell activation by running in p16 knockout dentate gyrus, differentially expressed in Set A_Set E_GLM selection

Here we focus mainly on the Set A_Set E_GLM selection, as this may represent a comprehensive ensemble of genes involved in the activation of stem cells by running.

### Analysis of the genes most upregulated in Set A_Set E_GLM

We analyze in more functional detail the genes most upregulated by running in p16 knockout dentate gyrus, in Set A_Set E_GLM, see Table 2.

#### Synaptic regulators

The following synaptic regulators are included: *Nlrc5*, required for MHC I expression, which regulates synapse plasticity (Li et al., 2021a; see also below its role as inhibitor of ROS levels); the Leptin receptor *Lepr*, which increases synaptogenesis by promoting the formation of mature spines and the activity of glutamate hippocampal synapses (Dhar et al., 2014), and whose ligand leptin stimulates dentate gyrus stem cells proliferation (Garza et al., 2008); *Clcn1*, voltage-dependent ClC-1 chloride channel that regulates chloride channels and is crucial for the propagation of the action potential (Imbrici et al., 2015); *Unc13d,* which is an essential element of the presynaptic vescicle fusion apparatus, controlling the fusion of synaptic vesicles with the plasma membrane (Dittman, 2019); *Slc18a2*, a vesicular monoamine transporter (VMAT) promoting presynaptic storage and release of neurotransmitters also in the hippocampus (Branco et al., 2020); *Cenpf*, a protein part of the N-cadherin-mediated synaptic adhesion apparatus, which connects pre- and postsynaptic membranes and regulates the efficiency of synaptic transmission (Tanaka et al., 2012) and also regulates chromosome segregation during mitosis (Zhou et al., 2019); *Dbn1* (Drebrin), which regulates memory activities by combining with or depolymerizing F-actin (Mizui et al., 2014), and plays a critical role in synaptogenesis and synaptic plasticity (Aoki et al., 2005); *Grm1*, the metabotropic glutamate receptor 1, whose activation may facilitate dentate gyrus neurogenesis, as shown in organotypic hippocampal slice cultures (Baskys et al., 2005); *Gabbr2*, the GABA B receptor 2, whose increase by running in p16 knockout dentate gyrus is consistent with the fact that the activation of GABA B receptors in the dentate gyrus is necessary for the development of LTP, in consequence of receptor-mediated disinhibition of other inhibitory processes (Heaney and Kinney, 2016).

#### Cell cycle regulators and promoters of stem cell proliferation

Upregulated cell cycle regulators or markers included in Set A_Set E_GLM comprise *Mki67* (Scholzen and Gerdes, 2000); the DNA topoisomerase II alpha *Top2a*, required for DNA replication (Gómez-Herreros et al., 2014), and necessary for neurogenesis in neurogenic niches (Qin et al., 2022); *Pole*, i.e., the DNA polymerase epsilon, catalytic subunit, involved in DNA replication and also recombination (Hogg and Johansson, 2012); *Prc1*, essential for successful cytokinesis and localization of the central spindle (Mollinari et al., 2002). Moreover, Set A_Set E_GLM includes also positive regulators of stem/progenitor cell proliferation such as *Zic4*, which activates the proliferation of progenitor cells in cerebellum (Blank et al., 2011); *Insm2*, required for glucose-stimulated insulin secretion (Wang et al., 2018); *Dpf3*, which is part of the SWI/SNF chromatin remodeling complex that promotes stem cells proliferation/self-renewal (Lessard et al., 2007); or, remarkably, *Trp73*, which plays a critical role in promoting self-renewal and proliferation of the neural stem and early progenitor cells, possibly through increasing Sox2 (Talos et al., 2010).

#### Negative regulators of ROS levels and oxidation

Negative regulators of ROS levels and oxidation whose expression is upregulated in SetA_Set E_GLM are: *Nlrc5*, which causes decrease of the ROS levels in hippocampal cells through Nrf2 (Li L. et al., 2021); *Mocos*, which decreases the ROS species during oxidative stress and is required during synaptogenesis (Féron et al., 2016); and *Gstm2*, which protects against dopamine oxidation through GSH-conjugation (Segura-Aguilar et al., 2014).

#### Regulator of fatty acid metabolism

*Lpin2* belongs to the Lipin family whose members act also as transcriptional coactivators that, in conjunction with *Pgc1-alpha* (peroxisome proliferator-activated receptor gamma, coactivator 1 alpha), control the expression of genes involved in lipid and mitochondrial metabolism. *Lpin 1* is required for the regulation of fatty acid metabolism, and *Lpin2* may play similar roles (Chen et al., 2015). In particular, Lipins favor lipid storage thanks to their phosphatidic acid phosphatase (PAP) enzymatic activity, which is involved in the generation of fatty acids through the production of triacylglycerols (Carman and Han, 2009). This is the first evidence of *Lpin2* expression in the hippocampus, whose increase by running may underlie the activation of stem cells by running. In fact, this is consistent with the observation that the activation of quiescent stem cells depends on a gradual decrease of the break down (i.e., oxidation) of fatty acids (Knobloch et al., 2017).

### Analysis of the genes most downregulated in Set A_Set E_GLM

The genes most downregulated by running in p16 knockout dentate gyrus, in Set A_Set E_GLM, are summarized below and listed in Table 2.

#### Neurotransmitter receptors

Downregulated neurotransmitter receptors include: *Chrm5,* acetylcholine receptor muscarinic 5, which is involved in presynaptic function (Sakata and Overacre, 2017); *Gabra3*, GABA(A) receptor alpha 3, which is of key importance for the activation of inhibitory GABA synapses (Wagner et al., 2021), and is remarkable that the decrease of *Gabra3* expression induced by running in p16 knockout dentate gyrus is in line with the notion that the inhibition of the GABA pathway favors stem cells activation (Dumitru et al., 2017). Another neurotransmitter receptor which is downregulated is *Grm3*, which is a metabotropic glutamate receptor (GluR) Group II, whose effect on adult neurogenesis is not clear, but the activation of GluR group II and III appears to have an inhibitory effect on neural progenitor cells proliferation (Jansson and Åkerman, 2014), and this is consistent with the decrease of expression of *Grm3* in p16 KO RUN dentate gyrus.

#### Synaptic plasticity regulators

*Rgs14* is a mitotic spindle protein that plays a role in synaptic plasticity, as it restricts calcium elevations in hippocampal spines (Evans et al., 2018), and whose deletion favors synaptic plasticity and LTP in the hippocampus (Lee et al., 2010). The inhibitory effect of *Rgs14* on synaptic transmission is also compatible with its downregulation observed in Set A (−0.38 log2 fold change) and Set D (−0.20 log2 fold change).

*Fgf12* is an intracellular factor involved in neurotransmission that interacts with voltage-gated sodium channels and regulates the channel activity in neurons (Zhang et al., 2012), however is not clear whether its decrease may facilitate stem cells activation.

*Nrxn1* is one of the neurexins, which are required for the localization and function of presynaptic GABA_B_-receptor signaling complexes (Luo et al., 2021); therefore, the decrease of *Nrxn1* expression negatively modulates the GABA pathway, which induces an expansion of the stem cells pool (Dumitru et al., 2017).

*Glrb* (Glycine receptor-β) is an inhibitory synaptic receptor, and this suggests that the decrease of *Glrb* may reduce its inhibition to the activation of neurogenesis by running in p16 knockout mice (Lee et al., 2017).

#### Regulators of cell cycle, neural migration and differentiation

A downregulated cell cycle regulator is *Klhl4*, a member of the Klhl protein family, which mediates the ubiquitination of interacting proteins and activates the transcription of p21, thus resulting in the inhibition of cell cycle (Choi et al., 2020); this would be consistent with the decrease of *Klhl4* expression induced by running in p16 knockout dentate gyrus. Another regulator is *Ttc9*, which interacts with tropomyosin, and since the primary function of tropomyosin is to stabilize actin filament, this interaction may play a role in cell shape and motility (Cao et al., 2008), with a possible role in stem/progenitor cells migration (Sun et al., 2015); *Ttc9* downregulation may thus suggest a decreased motility of exercise-activated stem/progenitor cells in the p16 knockout dentate gyrus.

Moreover, the gene *Ramp3* (receptor activity modifying protein 3), required for neuron differentiation (Li C. et al., 2021), is included among the genes downregulated in Set A and Set D, and this is consistent with the gene profile of stem cells proliferative activation that we are seeking to identify.

*In summary*, the above analysis focuses on genes that may play a causal role in the activation of neural stem cell. These include *Zic4*, which regulates progenitor cell proliferation, the negative regulators of ROS levels *Nlrc5*, *Gstm2* and *Mocos*, the regulator of fatty acid metabolism *Lpin2*, and the prodifferentiative gene *Ramp3* (downregulated in Set D; Li C. et al., 2021) or *Insm2*, required for the activation of the glycolitic pathway (Wang et al., 2018). The upregulation by running in p16 knockout dentate gyrus of the negative regulators of ROS levels *Nlrc5*, *Gstm2* and *Mocos*, in both Set A_Set E_GLM and Set D_Set E_GLM, is interesting, in view of the fact that a decrease of reactive oxygen species (ROS) accompanies the activation of stem cells by running (Adusumilli et al., 2021). As for *Lpin2*, the Lipin family has dual opposite actions on lipid metabolism, acting as a PAP (phosphatidate phosphatase) enzyme, required for lipid synthesis, and at the same time acting as a transcriptional coactivator promoting fatty acid oxidation. The balance of these two actions may contribute to lipid metabolic homeostasis (Chen et al., 2015), and this may be relevant since the control of fatty acid oxidation regulates neural stem cells activity (Knobloch et al., 2017). Among the synaptic regulators upregulated by running in both Set A_Set E_GLM and Set D_Set E_GLM, genes of interest are *Lepr*, *Slc18a2*, *Cenpf*, *Unc13d*, *Gabbr2*, and the synaptic inhibitor *Rgs14* that is downregulated, as they control synaptic plasticity and function, including release of neurotransmitters. Moreover, relevant for stem cell activation may also be the upregulation of cell cycle regulators, common to both Sets, such as *Top2a*, *Prc1*, *Pole* that are implicated in DNA replication and cytokinesis and may thus be involved in the proliferative activation of p16 knockout stem cells by running. Also *Cenpf* is interesting, as it plays a dual role in regulating the chromosome segregation as well as synaptic transmission.

Of note, the idea that Set A_Set E_GLM selection is a genuine and comprehensive collection of genes related to the activation of stem cells by running, is supported not only by their function profile related to the modulation of neural activity, but also from the observation that the genes differentially expressed in Set B comparison (KO CTL vs WT CTL, i.e., the genes representing the genetic signature of the p16 knockout phenotype), are a different subset, only marginally overlapping with Set A_Set E_GLM or with Set D_Set E_GLM selection (see section above “Genetic signature of the p16 knockout in the aged dentate gyrus (Set B comparison)”)*.*

### Repeated running stimuli fail to activate stem cell proliferation in p16 KO dentate gyrus as well as to induce differential expression of related genes

Finally, we asked whether the observed proliferative hyperactivation of p16 KO stem and progenitor cells by running made the cells prone to being reactivated by sequential running stimuli or had the opposite effect. We aimed to verify whether the stem cell-specific genes identified here play a role in the repeated response, and to gather information on the potential of stem cells self-renewal/proliferative activity.

**FIGURE 5:**
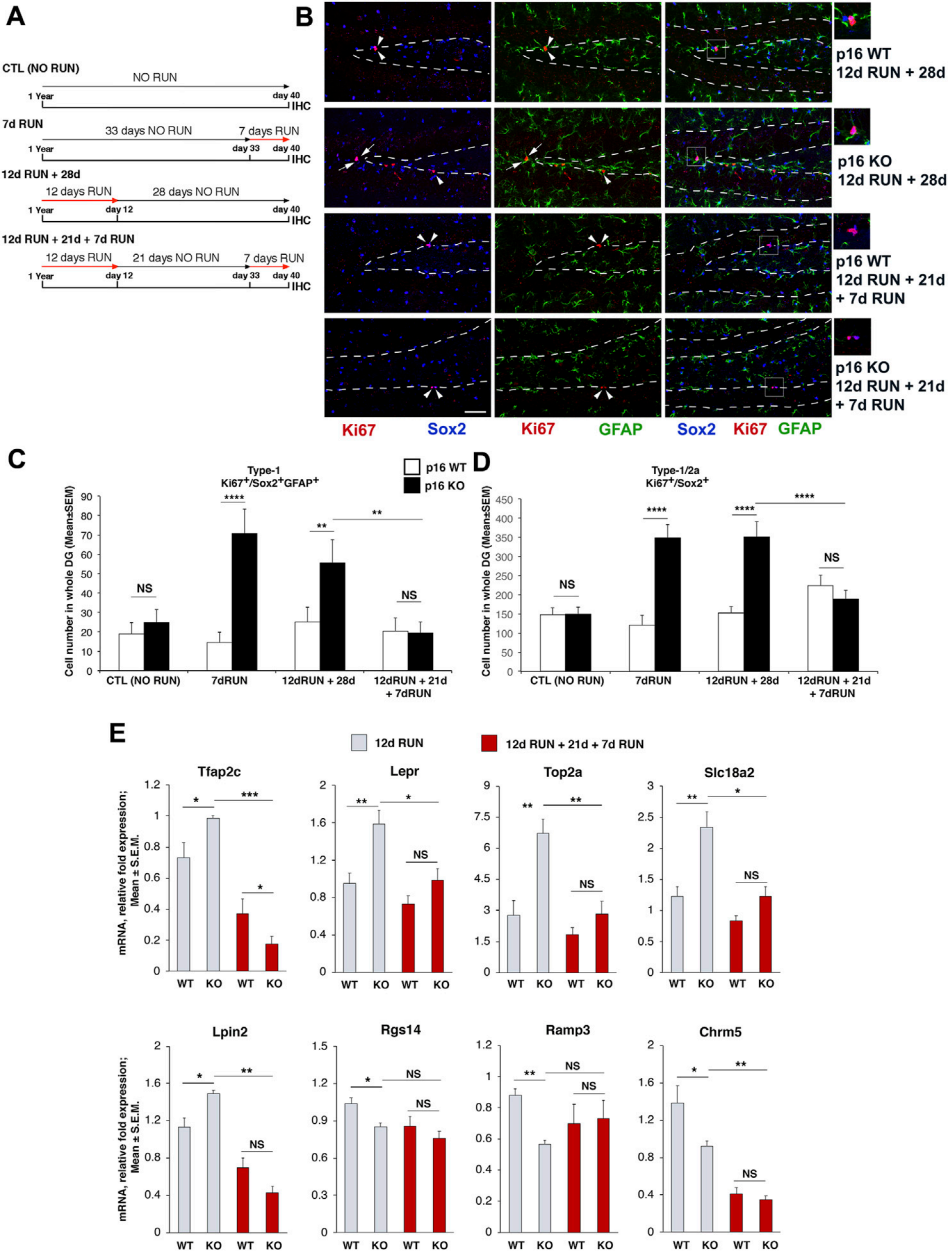
Cellular and gene expression analyses of stem cell activation elicited by sequential running stimuli in p16 KO and p16 WT dentate gyrus. **(A)** Timeline of the experiments performed on p16 WT and p16 KO 1-year-old mice: i) no run; ii) single run of 7 days; iii) single run of 12 days followed by a sedentary period; iv) double run of 12 and 7 days spaced 21 days apart. **(B)** Representative images by confocal microscopy (×40 magnification) showing that the proliferating type-1 stem cells (Ki67^+^/Sox2^+^/GFAP^+^, labeled in red, blue and green, respectively) in the dentate gyrus of p16 KO mice increase, relative to p16 WT, after a single run of 12 days followed by a sedentary period, whereas they do not increase after a double run of 12 and 7 days spaced 21 days apart. The white dashed line labels the boundaries of the dentate gyrus. Arrowheads: Ki67^+^/Sox2^+^/GFAP^−^ cells, arrows: Ki67^+^/Sox2^+^/GFAP^+^ cells. Scale bar 50 μm. White boxes: area at higher magnification (×2), shown in the panels on the right. **(C)** Graph showing the changes induced by running on the proliferation of stem cells (type-1 cells, Ki67^+^/Sox2^+^/GFAP^+^) in p16 WT and in p16 KO dentate gyrus, in the different groups indicated. A single RUN of 12 days induces the proliferation of p16 KO stem cells for an extended period (28 days; group 12d RUN +28d), but a second running added (7 days; group 12d RUN +21d + 7d RUN) does not stimulate p16 KO stem cell proliferation. **(D)** Similar changes were observed analyzing stem and progenitor cells together (i.e., type-1 and type-2a cells, respectively; Ki67^+^/Sox2^+^cells). **(C,D)** Analysis of simple effects: NS *p* > 0.05, **p < 0.01, or ****p < 0.0001, PLSD ANOVA test. Cell numbers in the dentate gyrus are means ± SEM of the analysis of five animals per group. **(E)** Real-time PCR analysis of the differential expression response to the second running stimulus for the Set A_Set E_GLM genes whose activation by running had been validated, i.e., *Tfap2c*, *Lepr*, *Top2a*, *Slc18a2*, *Lpin2*, *Rgs14*, *Ramp3*, *Chrm5*. The expression of these genes was activated in the p16 knockout dentate gyrus by a single run of 12 days but was not changed by a second running, relative to p16 wild-type. The figure shows the mean mRNA expression fold increases ±SEM from two independent experiments. Four biological replicates were used for mice that run 12 days and five biological replicates for mice that underwent the double run. TBP was used to normalize data. The statistical analysis of real-time PCR data was performed by non-parametric Kruskal-Wallis test to detect main effects (resulting with *p*-value <0.05 for all the genes tested), followed by Mann-Whitney U *post hoc* test for single Set comparison. *p < 0.05, **p < 0.01, ***p < 0.001, Mann-Whitney U *post hoc* test; n = at least 24, i.e., total number of data for each gene analyzed for all four groups (WT RUN and KO RUN, WT 12d RUN +21d + 7d RUN or KO 12d RUN +21d + 7d RUN).

To this purpose we set up an experimental protocol with two sequential running stimuli. One-year-old p16 KO and p16 WT mice were divided into four groups, namely,: i) sedentary mice (CTL); ii) a group that ran for 7 days (7d RUN); iii) a group allowed to run voluntarily for 12 days and sacrificed after 28 days (12d RUN +28d); iv) and a group that, 21 days after the first 12-day session of running, was submitted to a second running session of 7 days (12d RUN +21d + 7d RUN). The timeline of the experiment is described in Figure 5A.

First, we verified that 7 days of running was effective in stimulating the proliferation of stem cells in p16 KO, compared to p16 WT mice, as previously observed after a protocol of 12-day running (Micheli et al., 2019). We observed that proliferating stem cells (i.e., type-1, Ki67^+^/Sox2^+^/GFAP^+^) in p16 KO dentate gyri are highly induced after 7 days of running, showing 4.8-fold increase, relative to p16 WT (p16 KO 7d RUN vs p16 WT 7d RUN, *p* < 0.0001, Fisher’s PLSD ANOVA *post hoc* test; two-way ANOVA, interaction of running with genotype, *F*_(3,430)_ = 4.37, *p* = 0.0048 Figures 5A–C). As expected, no change is induced by running on p16 WT type-1 cell proliferation, relative to sedentary p16 WT (p16 WT 7d RUN vs p16 WT CTL, *p* = 0.71); likewise, sedentary p16 KO and p16 WT type-1 cell proliferation did not show differences (p16 KO CTL vs p16 WT CTL, *p* = 0.63). Of note, as we previously observed (Micheli et al., 2019), a large increase in type-1 cells proliferation was induced and was still present 28 days after the end of the 12-day running exercise (p16 KO 12d RUN +28d vs p16 WT 12d RUN +28d, 2.2-fold increase, *p* = 0.010, Fisher’s PLSD ANOVA *post hoc* test; Figures 5A–C). However, in the p16 KO group a second running exercise 7-day-long not only is ineffective on type-1 cells proliferation, relative to p16 WT (p16 KO 12d RUN +21d + 7d RUN vs p16 WT 12d RUN +21d + 7d RUN, *p* = 0.95, Fisher’s PLSD ANOVA *post hoc* test; Figures 5A–C) but also leads to a decrease with respect to the single-running group p16 KO 12d RUN +28d (*p* = 0.002, Fisher’s PLSD ANOVA *post hoc* test; Figures 5A–C). Similar results were obtained by analyzing together type-1 and type-2a stem and progenitor cells, respectively, i.e., Ki67^+^/Sox2^+^ (p16 KO 12d RUN +28d vs p16 WT 12d RUN +28d, 2.3-fold increase, *p* < 0.0001, Fisher’s PLSD ANOVA *post hoc* test; p16 KO 12d RUN +21d + 7d RUN vs p16 WT 12d RUN +21d + 7d RUN, *p* = 0.36, Fisher’s PLSD ANOVA *post hoc* test; Figures 5A, B, D). It is worth noting that also a second longer running exercise for 12 days did not increase type-1 cell proliferation (p16 KO 12d RUN +21d + 12d RUN vs p16 WT 12d RUN +21d + 12d RUN, data not shown).

Thus, the process of self-renewal of stem cells in p16 KO dentate gyrus appears to be long-lasting, relative to p16 WT, but with a limited capability of response to repeated stimuli. This suggest that a repeated stimulus in absence of p16 could lead stem cells to exit the cell cycle, thus favoring the maintenance of a pool of quiescent stem cells, or, alternatively, could cause a decrease of the stem cell pool. Additionally, we cannot exclude that the dentate gyrus stem cells may respond to a second stimulus if they are given more time to recover.

We also aimed to test the effect of a second running stimulus on the expression of the genes we identified in p16 KO as being correlated with the activation of stem cells after running, i.e., the Set A_Set E_GLM genes. Of this Set we analyzed the genes whose differential expression had been validated in this study, i.e., *Tfap2c*, *Lepr*, *Top2a*, *Slc18a2*, *Lpin2*, *Rgs14*, *Ramp3*, *Chrm5*. In spite of their observed regulation by the single 12-day run, (p16 WT RUN vs p16 KO RUN, *p*-value <0.05 for all genes, Mann Whitney U test, after analysis of main effects by Kruskal-Wallis test showing *p*-value <0.05; Figure 5E), their expression was not modified by the double-running protocol in the p16 knockout dentate gyrus, with respect to p16 wild-type, or, in the case of *Tfap2c,* it was even counter-regulated (p16 KO 12d RUN +21d + 7d RUN vs p16 WT 12d RUN +21d + 7d RUN; *Tfap2c*, *p* = 0.041; *Lepr*, *p* = 0.17; *Top2a*, *p* = 0.25; *Slc18a2*, *p* = 0.07; *Lpin2*, *p* = 0.07; *Rgs14*, *p* = 0.14; *Ramp3*, *p* = 0.91; *Chrm5, p* = 0.46; Mann Whitney U test; see Figure 5E). Moreover, comparing the gene expression level in p16 KO after the single 12-day run and after the double run, we found a significant reduction in 6 genes after the double run (p16 KO 12d RUN vs p16 KO 12d RUN +21d + 7d RUN; *Tfap2c*, *p* = 0.0004; *Lepr*, *p* = 0.018; *Top2a*, *p* = 0.004; *Slc18a2*, *p* = 0.018; *Lpin2*, *p* = 0.004; *Chrm5, p* = 0.004; Mann Whitney U test).

Thus, the lack of proliferative activation of type-1 stem cells in p16 knockout mice after the second stimulus matches the lack of differential expression, further suggesting the involvement of these Set A_Set E_GLM genes in the process of stem cell response to stimulus.

Indeed, in recent years two views have been proposed about how the stem cell pool in the subgranular zone of the dentate gyrus self-renews; one suggesting recurrent stem cell self-renewal, while the other configuring a “disposable stem cell” model.

In the first model, a quiescent stem cell that has been activated under physiological conditions may go through repeated asymmetrical divisions, generating neurons or astroglia. However, stem cells may also divide symmetrically and, following either type of division, return to a quiescent state, remaining open to activation in the future (Bonaguidi et al., 2011). Conversely, according to the “disposable stem cell” paradigm, the stem cell is activated, divides multiple times asymmetrically, and then definitively differentiates into an astrocyte or a neuron, depleting the pool (Encinas et al., 2011). However, if the activation is induced by a stimulus of strong intensity (kainic acid), which mimics epileptiform activity, then the division mode changes from asymmetric to symmetric with prevalent astrocytogenesis and enhanced pool depletion (Sierra et al., 2019).

Moreover, according to other studies, the retention of the stem cell pool and neurogenesis in old age may be caused by the fact that not all neural stem cells are rapidly exhausted and that some of them return to quiescence in both the subgranular zone (SGZ) and the SVZ (Urbán et al., 2016; Obernier et al., 2018; Pilz et al., 2018).

Pilz’s et al. (2018) model actually suggests that radial glia stem cells may enter the cell cycle infrequently and alternate between quiescence and activity. According to Urbán et al. (2016) the activated dentate gyrus stem cells can in the long-term revert to a resting state if the E3-ubiquitin ligase Huwe1 is destroyed, which eventually prevents the upregulation of cyclin D1. Indeed, stem cell returning to a quiescent state is a necessary step for the long-term maintenance of hippocampus neurogenesis, since the proliferative stem cell pool is depleted when stem cells fail to enter quiescence (Urbán et al., 2016).

More recently, the two views have been united by recognizing the importance of age in stem cell self-renewal. In fact, at an early age the second model would be favored, with intense replication followed by depletion of the pool, whereas at later ages and during aging stem cells would replicate according to a gradual pattern of self-renewal that favors the conservation of the pool; this age-associated change is parallel to a shift from a population of stem cells dividing repeatedly to a more quiescent population (Harris et al., 2021; Ibrayeva et al., 2021; Martín-Suárez and Encinas, 2021).

We showed that the proliferation of p16 knockout stem cells (Ki67^+^/Sox2^+^/GFAP^+^) remains active after the first running stimulus, relative to p16 wild-type mice, remarkably even 28 days after the end of the exercise. However, after a second neurogenic stimulus (i.e., 7-day running), the stem cell pool appears unresponsive to stimulus, since proliferating stem cells of p16 knockout dentate gyrus do not display a significant change relative to p16 wild-type, and are even reduced relative to the p16 knockout group undergoing a single running exercise, (i.e., p16 KO RUN 12d + 28d), reaching the level of sedentary mice. This suggests that a protracted proliferation after the end of the first running, in a condition lacking the inhibitory control of p16 expression, may reduce the response potential of stem cells. This effect may mean a reduced number of responsive stem cells remaining 3 weeks after the first running stimulus; however, although this points to the safeguarding effect of p16 on the cell pool functional integrity, our data do not directly suggest that a depletion of the stem cell pool has occurred. Our transcriptomic analysis monitors the genetic response of p16 knockout at the end of the first (12-day) running stimulus. Notably, the match between the lack of responsiveness of the Set A_Set E_GLM genes to the second run and the lack of proliferative activation of p16 knockout stem cells is consistent with our idea that this gene set plays a role in stem cells activation after the first running stimulus and in the non-activation after the second stimulus. If so, the modulation of those p16-dependent genes could ensure that the stem cell pool is preserved during aging.

## Conclusion

The main cellular phenotypes that we have observed previously in the p16 KO dentate gyrus, correspond to i) an increase of apoptosis relative to p16 WT either sedentary or running mice, and to ii) a strong activation of stem and progenitor cells induced by running (Micheli et al., 2019). Consistently, here we find that the genes most significantly upregulated by p16 knockout in the dentate gyrus of sedentary mice (Set B, i.e., the basic p16 KO gene signature not associated with running) are involved in apoptosis and neuroinflammation (*Eif2ak2, Ccr1, Cd59a*), in cell signaling, metabolism and synaptic neuroplasticity and activity (lactate transport, NOS), suggesting a reactive cellular state, ready to respond to external stimuli. In fact, the GO classes enriched in these Set B genes include response to endogenous stimulus, regulation of signaling, cell cycle, and response to oxygen containing compounds.

On the other hand, the 106 differentially expressed genes of Set A_Set E_GLM selection, identified as correlated to the activation of stem cells by running in p16 KO dentate gyrus, appear to be an ensemble relatively different from Set B genes, and are involved in synaptogenesis and synaptic function, glutamate and GABA metabolism, cell cycle control and promotion of stem cell proliferation, regulation of ROS levels and oxidation as well as neurotransmitter activity. The differential changes of expression of these genes, either up-or downregulated by running, appear to correspond to an activation of these processes, in line with their correlation to a phenotype of proliferative activation of stem and progenitor cells.

Further studies will be necessary to ascertain whether the up- or downregulation in the dentate gyrus of one or more of the genes belonging to Set A_Set E_GLM selection is sufficient by itself to stimulate stem cells activation or self-renewal in wild-type sedentary mice. It would also be interesting to verify whether the same gene patterns differentially regulated by running are observed in a conditional mouse model that ablates p16 selectively in the hippocampus or in stem cells.

The cohort of the Set A_Set E_GLM stem cell-specific genes, activated by 12-day running in p16 knockout dentate gyrus, may also support the observed enhancement of stem cell proliferation still ongoing 1 month after the termination of the exercise. Moreover, parallel to the inability of the second running stimulus to induce stem cell proliferation in p16 knockout dentate gyrus, the expression of several Set A_Set E_GLM genes is not modified, and this could be at the origin of the lack of stem cell activation by the second run, being conceivably in line with the idea that these genes are responsible for the stem cell pool activation and maintenance during aging. In particular, in keeping with the self-renewal model favoring quiescence during aging, proposed by Harris et al. (2021) and by Ibrayeva et al. (2021), Set A_Set E_GLM genes may play a role as regulators in stem cells of the shift from activity to quiescence and viceversa.

## Figures and Tables

**Table:** 

**ANOVA**	analysis of variance
**Btg1**	B-cell translocation 1 gene
**DE**	differentially expressed
**DG**	dentate gyrus
**FDR**	false discovery rate
**GFAP**	glial fibrillary acidic protein
**DCX**	doublecortin
**NeuN**	neuronal nuclei
**GO**	Gene Ontology
**Gsea**	Gene Set Enrichment Analysis
**GOCC**	Gene Ontology Cellular Components
**GOMF**	Gene Ontology Molecular Functions
**GLM**	Generalized Linear Model
**LRT**	likelihood ratio test
**MSigDB**	Molecular Signatures Database
**RT-PCR**	real-time PCR
**TBP**	TATA-binding protein
**GOBP**	Gene Ontology biological processes
**KO**	knockout
**PFA**	paraformaldehyde
**PBS**	phosphate buffered saline
**PLSD**	protected least significant difference
**ROS**	reactive oxygen specie
**SEM**	standard error of the mean
**SGZ**	subgranular zone
**Sox2**	sex determining region Y-box 2
**SVZ**	subventricular zone
**TRITC**	tetramethylrhodamine isothiocyanate
**WT**	wild-type
**Ccr1**	C-C motif chemokine receptor 1
**Chrm5**	cholinergic receptor muscarinic 5
**Eif2ak2**	eukaryotic translation initiation factor 2 alpha kinase 2
**Lepr**	leptin receptor
**Lpin2**	lipin2
**Magt1**	magnesium transporter 1
**Ntsr2**	neurotensin receptor 2
**Ramp3**	receptor activity modifying protein 3
**Rgs14**	regulator of G protein signaling 14
**Slc16a1**	solute carrier family 16 member 1
**Slc18a2**	solute carrier family 18 member A2
**Tfap2c**	transcription factor AP-2 gamma
**Top2a**	DNA topoisomerase II alpha
**Lpl**	lipoprotein lipase
**PAP**	phosphatidic acid phosphatase

## Data Availability

The datasets presented in this study can be found in online repositories. The names of the repository/repositories and accession number(s) can be found below: https://www.ncbi.nlm.nih.gov/geo/query/acc.cgi?acc=GSE237736.
