# Supplementary figures and images for: Transcriptome analysis reveals genes associated with stem cell activation by physical exercise in the dentate gyrus of aged p16Ink4a knockout mice
Source: Front Cell Dev Biol. 2023 Oct 19;11:1270892. doi: 10.3389/fcell.2023.1270892 (PMC10621069; doi:10.3389/fcell.2023.1270892)

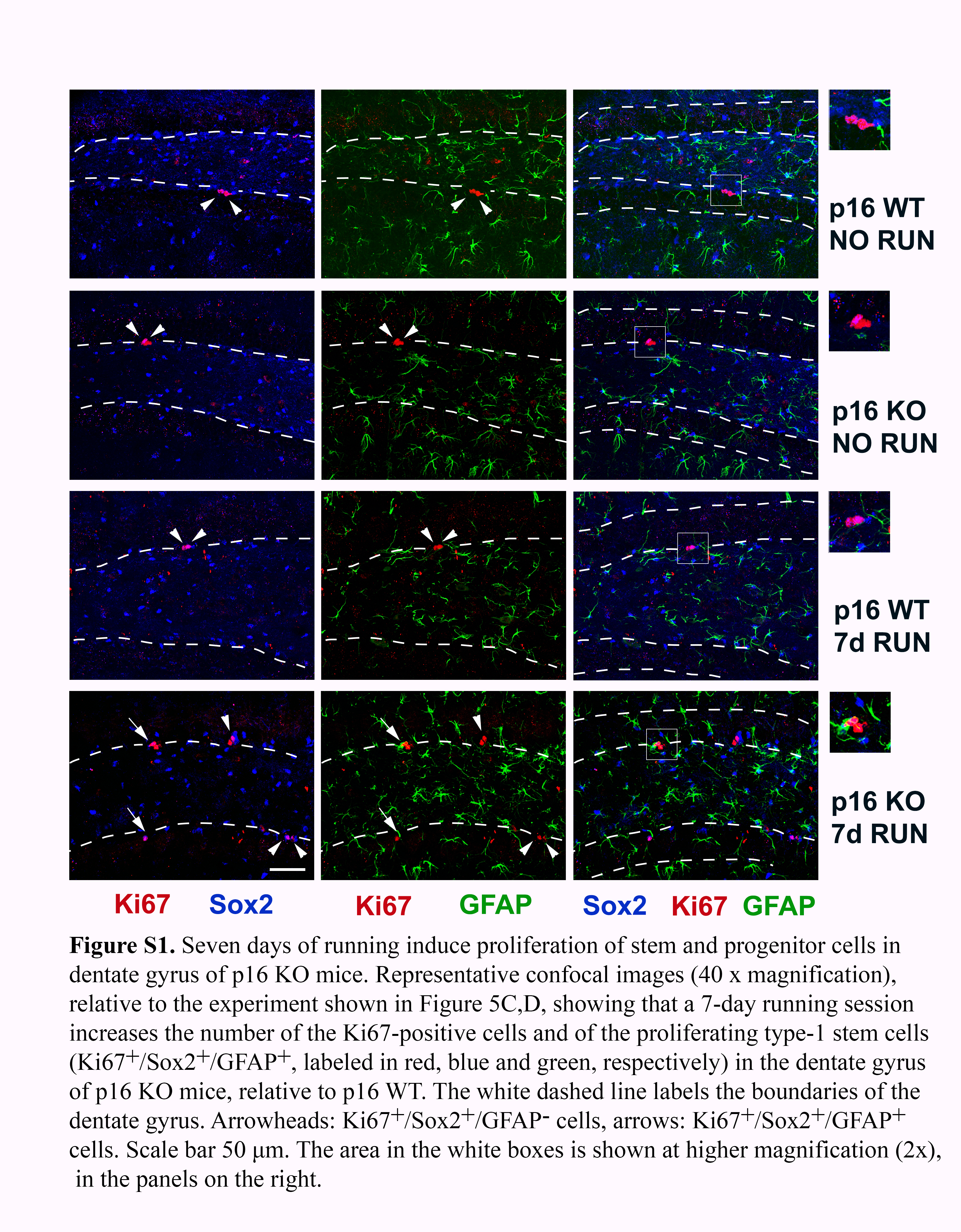

Supplement: Supplementary file 3 [file Image1.TIF]
